# T-type Ca^2+^ and persistent Na^+^ currents synergistically elevate ventral, not dorsal, entorhinal cortical stellate cell excitability

**DOI:** 10.1016/j.celrep.2023.112699

**Published:** 2023-06-26

**Authors:** Aleksandra Topczewska, Elisabetta Giacalone, Wendy S. Pratt, Michele Migliore, Annette C. Dolphin, Mala M. Shah

**Affiliations:** 1 Pharmacology, School of Pharmacy, University College London, London WC1N 4AX, UK; 2 Institute of Biophysics, National Research Council, 90146 Palermo, Italy; 3 Neuroscience, Physiology and Pharmacology, University College London, London WC1E 6BT, UK

**Keywords:** medial entorhinal cortex, stellate cells, ion channels, calcium, intrinsic activity, synaptic potentials

## Abstract

Dorsal and ventral medial entorhinal cortex (mEC) regions have distinct neural network firing patterns to differentially support functions such as spatial memory. Accordingly, mEC layer II dorsal stellate neurons are less excitable than ventral neurons. This is partly because the densities of inhibitory conductances are higher in dorsal than ventral neurons. Here, we report that T-type Ca^2+^ currents increase 3-fold along the dorsal-ventral axis in mEC layer II stellate neurons, with twice as much Ca_V_3.2 mRNA in ventral mEC compared with dorsal mEC. Long depolarizing stimuli trigger T-type Ca^2+^ currents, which interact with persistent Na^+^ currents to elevate the membrane voltage and spike firing in ventral, not dorsal, neurons. T-type Ca^2+^ currents themselves prolong excitatory postsynaptic potentials (EPSPs) to enhance their summation and spike coupling in ventral neurons only. These findings indicate that T-type Ca^2+^ currents critically influence the dorsal-ventral mEC stellate neuron excitability gradient and, thereby, mEC dorsal-ventral circuit activity.

## Highlights

• T-type Ca_V_3.2 Ca^2+^ currents are 3-fold larger in ventral than dorsal mEC stellate neurons
• T-type Ca^2+^ and persistent Na^+^ currents boost membrane depolarization in ventral neurons
• Consequently, firing frequency is elevated in ventral neurons compared with dorsal neurons
• T-type Ca^2+^ currents enhance EPSP-spike coupling in ventral, not dorsal, stellate neurons

## Graphical abstract

**Figure:**
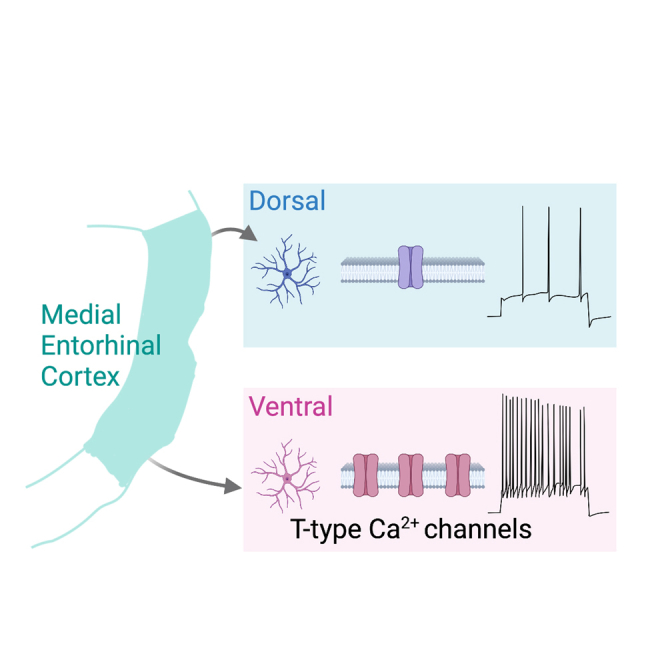

## Teaser

Topczewska et al. show that there is a three-times-greater T-type Ca^2+^ current amplitude in ventral medial entorhinal cortical layer II stellate neurons compared with their dorsal counterparts. These currents interact with persistent Na^+^ currents to elevate action potential firing frequency and EPSP-spike coupling in ventral stellate neurons only.

## Introduction

The medial entorhinal cortex (mEC) forms a significant component of the hippocampal formation and critically influences episodic memory formation, spatial memory, and spatial navigation.^1^ There is mounting evidence that the mEC, like the hippocampus proper, can support such diverse functions by having within-cell-type heterogeneity, which enables distinct computations to be executed.^2^ Certainly, mEC stellate layer II neuron membrane properties and, consequently, firing frequency vary along the dorsal-ventral axis, with dorsal neurons displaying fewer action potentials than ventral neurons in response to depolarizing stimuli. Accordingly, there is a dorsal-ventral expansion of the size and spacing of neuronal ensembles, grid cells, that encode information about our location within the environment.^1^ Since dorsal and ventral mEC layer II stellate neurons connect to dorsal and ventral hippocampal neurons respectively, altered dorsal-ventral mEC neuron activity and grid cell firing patterns will influence the corresponding hippocampal neurons and place cell firing, neural network activity associated with spatial navigation and memory.^1^^,^^3^^,^^4^ Hence, understanding the cellular mechanisms regulating spike firing rates in dorsal and ventral mEC stellate neurons will enhance our knowledge of the factors contributing to the generation and properties of grid cells, which in turn will lead to a better understanding of processes such as spatial memory formation.

Voltage-gated ion channels are critically involved in the generation and propagation of action potentials and synaptic potentials.^5^^,^^6^ Their expression varies along the mEC dorsal-ventral axis^2^^,^^7^ and is correlated with differences in ion channel currents observed in mEC layer II stellate neurons. Voltage-gated K^+^ and hyperpolarization-activated cyclic nucleotide-gated (HCN) channel densities are higher in dorsal neurons than their ventral counterparts.^8^^,^^9^^,^^10^ Remarkably, there is a similar variation in these channel densities in hippocampal CA1 pyramids.^11^^,^^12^^,^^13^ As in hippocampal CA1 pyramids, the elevated K^+^ and HCN channel presence in dorsal mEC stellate neurons renders them less excitable compared with their ventral counterparts.^8^^,^^11^^,^^12^^,^^14^^,^^15^ It is, though, unknown whether ventral mEC stellate neurons express particular ion channel conductances that preferentially and actively enhance their intrinsic and synaptic activity compared with dorsal neurons.

T-type Ca^2+^ channels are of interest as they influence neuronal activity and synaptic plasticity.^16^^,^^17^ These channels activate at voltages above the resting membrane potential (RMP) and inactivate within tens of milliseconds.^17^^,^^18^^,^^19^ Consistent with their biophysical properties, T-type Ca^2+^ currents promote low threshold Ca^2+^ spikes and so-called burst firing (i.e., a brief clustering of action potential) in many neurons, including hippocampal CA1 pyramids and thalamic neurons.^16^^,^^17^^,^^18^^,^^19^^,^^20^ T-type Ca^2+^ currents are present in mEC layer II stellate neurons,^21^^,^^22^ although little is known about how they affect their intrinsic membrane and action potential properties. It is also unknown if the density of these currents varies along the mEC dorsal-ventral axis.

Here, we report that T-type Ca^2+^ current amplitudes were three times larger in mEC ventral stellate neurons compared with dorsal neurons. The expression of the T-type Ca^2+^ channel subunit, Ca_V_3.2, in ventral mEC was also twice that in dorsal mEC. T-type Ca^2+^ currents prolonged EPSPs, thereby enhancing the summation of EPSP trains and EPSP-spike coupling in ventral, and not dorsal, stellate neurons. Additionally, these currents acted in concert with subthreshold Na^+^ currents to preferentially augment ventral stellate neuron membrane resistance measured using depolarizing stimuli and, consequently, tonic action potential firing. This is an unusual mechanism by which T-type Ca^2+^ channels raise excitability in central neurons. As T-type Ca_V_3.2 Ca^2+^ currents are predominantly located in ventral neurons, these findings suggest that manipulation of their activity will yield insights into the role of ventral stellate neurons in mEC circuit activity and functions.

## Results

### T-type Ca_V_3.2 Ca^2+^ currents enhance wildtype ventral, not dorsal, stellate neuron action potential firing propensity

**Figure 1:**
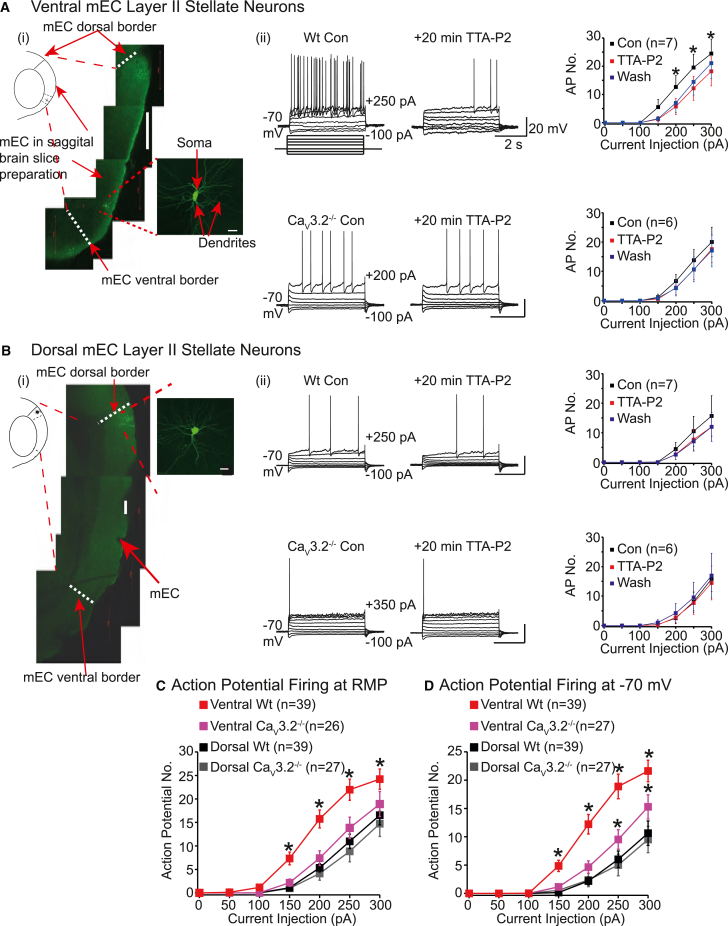
Ca_V_3.2 Ca^2+^ currents raise ventral, not dorsal, stellate neuron excitability (A and B) Schematics illustrating ventral (A(i)) and dorsal (B(i)) mEC regions respectively. Representative confocal brain slice images containing dorsal and ventral mEC layer II stellate neurons are shown (see Figure S1), with the morphologies of these neurons displayed in the insets. The scales in (A(i)) and (B(i)) represent 500 μm and 200 μm respectively, while those in the insets depict 20 μm. Typical electrophysiological recordings from (A(ii)) wild-type (WT) and (B(ii)) Ca_V_3.2^−/−^ neurons when the protocol shown was applied under control conditions (con) and after TTA-P2 application. The membrane potential is noted next to each trace. The scale associated with the first panel applies to all panels. The graphs on the right portray the mean ± SEM action potentials (AP no.) elicited by applying current injections in the absence (con), presence, and following washout of TTA-P2 in seven dorsal and ventral WT neurons and six dorsal and ventral Ca_V_3.2^−/−^ neurons as indicated in brackets. Paired t tests were used to determine significance (p) values (exact values in Table S7). (C and D) Average ± SEMs action potential numbers elicited by varying current amplitudes at RMP and −70 mV respectively in 39 dorsal and ventral WT neurons and 26 or 27 dorsal and ventral Ca_V_3.2^−/−^ neurons as indicated in parenthesis on the graphs. To determine significance, data from all groups were compared using a two-way ANOVA with Fisher’s least significance difference (LSD) *post hoc* test (exact p values are stated in Table S7). Asterisks signify significance at p < 0.05 in all graphs in the figure.

T-type Ca^2+^ channels are encoded by 3 α1 subunits: Ca_V_3.1, Ca_V_3.2, and Ca_V_3.3 ^17^. Ca_V_3.2 is predominantly expressed in the mEC.^23^ To determine how T-type Ca^2+^ currents influence dorsal and ventral mEC layer II stellate neuron activity, we made electrophysiological recordings from neurons present in parasagittal wildtype and Ca_V_3.2^−/−^ brain slices (see STAR Methods). Neurons were ascertained as being dorsal or ventral if they were located within 0%–30% or 70%–100% of the mEC-parasubiculum border respectively^24^ (Figures 1A(i), 1B(i), and S1; see STAR Methods). Stellate neurons were visually identified as having a polygonal or ovoid soma with multiple radiating dendrites (Figures 1A(i), 1B(i), and S2A). Full morphological analysis revealed no differences between dorsal and ventral wild-type and Ca_V_3.2^−/−^ neuron dendrite numbers (Figure S2B). Consistent with previous reports,^8^ the cell body area, though, was greater in dorsal than ventral wild-type and Ca_V_3.2^−/−^ neurons (Figure S2C).

Whole-cell current-clamp recordings showed that depolarizing steps induced significantly lower numbers of action potentials in ventral Ca_V_3.2^−/−^ neurons compared with wild types (Figures 1A(ii), 1C, and 1D). Both wild-type and Ca_V_3.2^−/−^ neurons, though, exhibited similar firing patterns (Figure 1A), with clusters of spikes being elicited.^14^ Further, 20-min application of the T-type Ca^2+^ channel inhibitors, 100 nM TTA-P2 or 50 μM NiCl_2_, onto ventral wild-type, but not Ca_V_3.2^−/−^, neurons significantly reduced the number of action potentials induced by depolarizing pulses (Figure 1A(ii) and S3). This effect was partially reversible upon 20-min washout of NiCl_2_ (Figure S3), but not TTA-P2 (Figure 1A(ii)). This accords with the slow reversal of the effects of TTA-P2 on T-type Ca^2+^ current in other neurons.^25^^,^^26^ In contrast to ventral neurons, neither the loss of Ca_V_3.2 subunit expression nor T-type Ca^2+^ channel inhibitors affected dorsal stellate neuron action potential numbers elicited with depolarizing pulses (Figure 1B(ii), 1C, 1D, and S3). Altogether, these findings indicated that T-type Ca_V_3.2 Ca^2+^ currents preferentially promote tonic, long-lasting action potential firing in ventral neurons and these currents influence the spike firing gradient along the dorsal-ventral axis in mEC stellate neurons.

### Ca_V_3.2 currents mask the membrane potential sag and increase R_N_ measured with depolarizing potentials in ventral wild-type stellate neurons to enhance excitability

As Ca_V_3.2 Ca^2+^ currents are fast inactivating,^27^ we asked how these currents might enhance action potential firing in ventral neurons. Neither abolition of Ca_V_3.2 subunits nor T-type Ca^2+^ channel inhibitors altered the RMP in ventral neurons, suggesting that these currents are not active at rest (Tables S1 and S2). Further, loss of Ca_V_3.2 subunits or application of T-type Ca^2+^ channel blockers did not alter the time taken for the first spike to be initiated with a depolarizing step, the afterhyperpolarization amplitude following a spike (Tables S1 and S2), or the shape or threshold of a single spike (Figure S4; Table S3).

**Figure 2:**
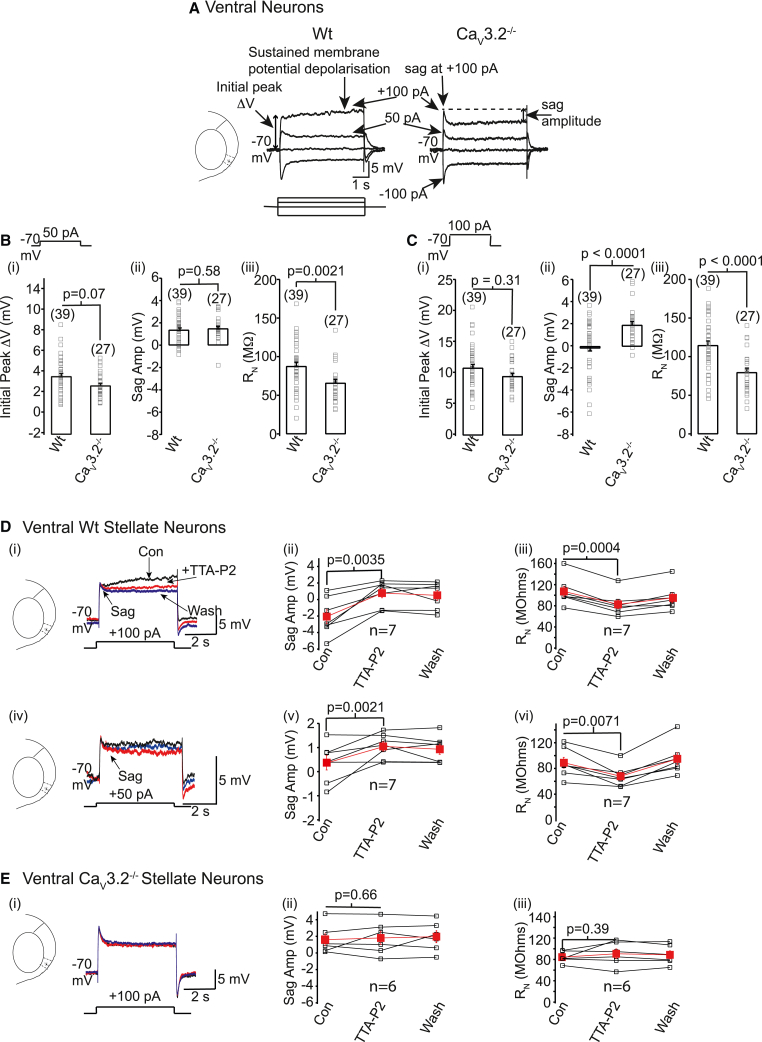
Ca_V_3.2 Ca^2+^ currents mask the membrane potential sag and enhance R_N_ at depolarizing potentials in ventral WT neurons (Ai) Example recordings obtained from WT and Ca_V_3.2^−/−^ neurons when the protocol shown was applied. The scale applies to both traces in the panel. (B(i)–C(iii)) Individual (open squares) and average ±SEM (bars) (B(i) and C(i)) initial peak voltage change (ΔV), (B(ii) and C(ii)) sag amplitudes (amp), and (B(iii) and C(iii)) R_N_ values obtained with +50 pA (B) and +100 pA (C) steps in 39 WT and 27 Ca_V_3.2^−/−^ neurons. Significance (p) values were obtained using a two-way ANOVA test corrected for multiple comparisons using a Bonferroni constant. (D and E) (D(i), D(iv), E(i)) Representative subthreshold depolarizing traces under control conditions (black), in the presence of TTA-P2 (red) and following washout of TTA-P2 (blue) in WT and Ca_V_3.2^−/−^ neurons. (D(ii), D(iii), D(v), D(vi), E(ii), E(iii)) Individual (black squares) and mean ± SEM (filled red squares) values of the sag amplitude and R_N_ obtained with subthreshold depolarizing steps under control conditions, with TTA-P2 and after washout in seven WT and six Ca_V_3.2^−/−^ neurons. Paired t tests were performed to obtain significant (p) values.

Increases in input resistance (R_N_) enhance action potential firing in stellate neurons.^8^^,^^14^ We therefore explored whether T-type Ca^2+^ currents affect R_N_ in ventral neurons. R_N_ measured with hyperpolarizing steps and the sag potential amplitude elicited with hyperpolarizing pulses (due to HCN channel activation) were not different between wild-type and Ca_V_3.2^−/−^ neurons (Figure 2A; Table S1). In contrast, R_N_ measured with +50 pA and +100 pA steps was significantly greater in wild types compared with Ca_V_3.2^−/−^ neurons (Figure 2; Table S1). The difference in R_N_ between wild-type and Ca_V_3.2^−/−^ neurons was less with +50 pA than +100 pA steps (Figure 2), which is consistent with greater Ca_V_3.2 Ca^2+^ current activation with increasing depolarization.^27^ The initial peak voltage change produced by subthreshold depolarizing pulses was similar in wild-type and Ca_V_3.2^−/−^ neurons (Figure 2). The sag potential amplitude elicited with +100 pA steps, and not +50 pA steps, was significantly reduced in wild-types compared with Ca_V_3.2^−/−^ neurons (Figure 2; Table S1). This suggests that the initial voltage jump generated with large (+100 pA) subthreshold depolarizing steps activated T-type Ca_V_3.2 Ca^2+^ currents in ventral wild-type neurons to mask the sag and instigate a prolonged membrane potential depolarization to enhance R_N_ (Figure 2A).

Further, TTA-P2 or NiCl_2_ applied onto wild-type, but not Ca_V_3.2^−/−^, neurons reduced R_N_ measured using depolarizing steps (Figures 2 and S3; Table S2). Neither compound had any effect on the initial peak voltage in wild-type or Ca_V_3.2^−/−^ neurons (Table S2). Moreover, TTA-P2 or NiCl_2_ application revealed a distinct sag with subthreshold depolarizing steps in wild-type, but not Ca_V_3.2^−/−^, neurons (Figures 2D, 2E, and S3). The sag amplitude change in wild-type neurons with +50 pA steps (0.56 ± 0.14 pA, n = 13) was significantly less than with +100 pA (2.28 ± 0.47 pA, n = 13, p = 0.0006, two-tailed paired t test), as expected with the voltage-dependent activation of T-type Ca^2+^ currents.^27^ The sag amplitude and R_N_ measured using hyperpolarizing potentials were unaffected by T-type Ca^2+^ channel inhibitors in wild-type or Ca_V_3.2^−/−^ neurons (Table S2). These findings, therefore, reinforce the notion that Ca_V_3.2 Ca^2+^ channels are activated with subthreshold depolarizing potentials in ventral wild-type neurons to mask the membrane potential sag, cause a prolonged depolarization, and enhance R_N_.

### Ca_V_3.2 currents significantly influence the mEC stellate neuron R_N_ dorsal-ventral gradient at positive potentials

**Figure 3:**
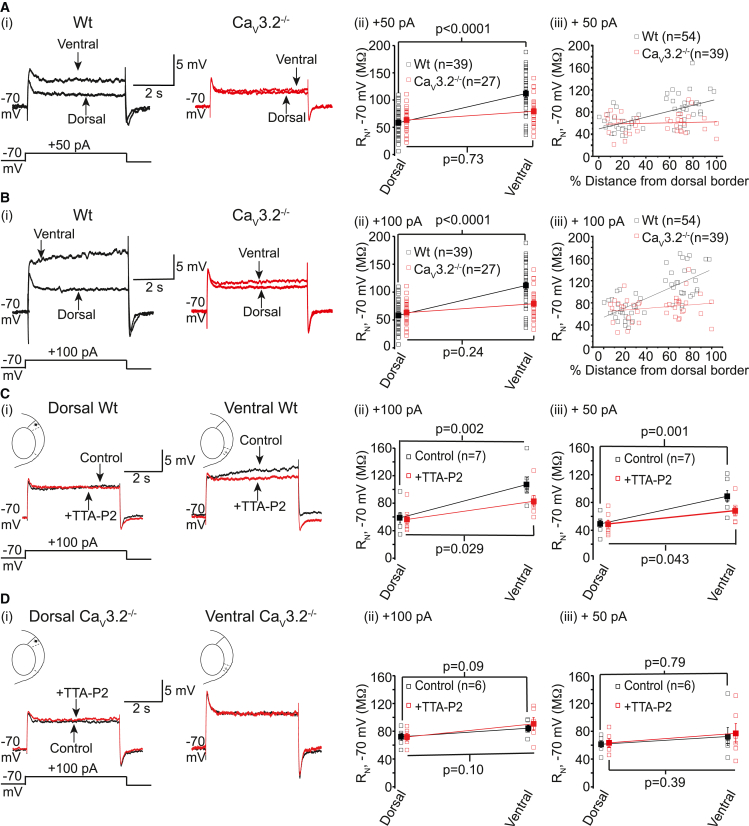
Ca_V_3 currents significantly influence the depolarizing R_N_ dorsal-ventral gradient in mEC stellate neurons (A and B) (A(i) and B(i)) Representative traces from WT and Ca_V_3.2^−/−^ neurons when subthreshold depolarizing current steps were applied. The scale shown applies to all traces within the panel. (A(ii) and B(ii)) Individual (open squares) and mean ± SEM (closed squares) R_N_ values from 39 WT (black) and 27 Ca_V_3.2^−/−^ (red) neurons. Statistical significance for comparison of ventral and dorsal neurons was determined using a two-way ANOVA corrected for multiple comparisons using a Bonferroni constant. (A(iii) and B(iii)) Individual WT (black) or Ca_V_3.2^−/−^ (red) R_N_ values plotted against their locations from the dorsal border (i.e., mEC-parasubiculum border). Linear regression analysis was carried out to obtain the Pearson’s correlation coefficients. (C(i) and D(i)) Example traces obtained from WT and Ca_V_3.2^−/−^ neurons respectively in the absence (control) and presence of TTA-P2. The scale shown in each panel applies to both traces. (C(ii), D(ii), C(iii), and D(iii)) Individual (open squares) and mean ± SEM (closed squares) R_N_ values obtained under control conditions (black) and withTTA-P2 (red) from seven WT and six Ca_V_3.2^−/−^ neurons. Unpaired t tests were employed to determine significance values.

Next, we asked if Ca_V_3.2 Ca^2+^ current activation in dorsal neurons alters R_N_, even though these currents had little effect on spike numbers elicited by depolarizing potentials. There was no difference in dorsal wild-type and Ca_V_3.2^−/−^ neuron R_N_, initial peak voltage, or membrane potential sag measured with depolarizing or hyperpolarizing steps (Figure 3; Table S1). Moreover, neither TTA-P2 nor NiCl_2_ had any effect on these parameters in wild-type and Ca_V_3.2^−/−^ neurons (Figures 3 and S3; Table S2). Thus, these results suggest that Ca_V_3.2 currents have no effect on dorsal neuron membrane properties.

Consistent with the biophysical properties of Ca_V_3.2 currents, R_N_ measured using subthreshold depolarizing steps was significantly greater in ventral than dorsal wild-type neurons (Figure 3; Table S1). This variation in R_N_ correlated with stellate neuron location along the dorsal-ventral axis (Pearson’s constant for correlation, *r* = 0.58 and 0.71 for R_N_ measured with +50 pA; Figure 3A(iii) and +100 pA Figure 3B(iii), respectively). This concurs with previously published results.^8^ This dorsal-ventral gradient in R_N_, though, was abolished in Ca_V_3.2^−/−^ stellate neurons (*r* = 0.07 and 0.19 for R_N_ measured with +50 pA and +100 pA respectively; Figure 3A(iii) and 3B(iii)). Moreover, this dorsal-ventral R_N_ gradient was significantly reduced in the presence of TTA-P2 in wild-type neurons (Figure 3C) as TTA-P2 caused a significant decrease in R_N_ measured using depolarizing pulses in ventral, but not dorsal, neurons (Figure 3C). TTA-P2 had little effect on dorsal-ventral R_N_ difference in Ca_V_3.2^−/−^ neurons measured with subthreshold depolarizations (Figure 3D). Altogether these findings support the notion that T-type Ca_V_3.2 Ca^2+^ currents critically influence the dorsal-ventral gradient in R_N_ at depolarizing potentials in stellate neurons.

### Ca_V_3.2 Ca^2+^ currents are predominantly located in ventral wild-type stellate neurons

**Figure 4:**
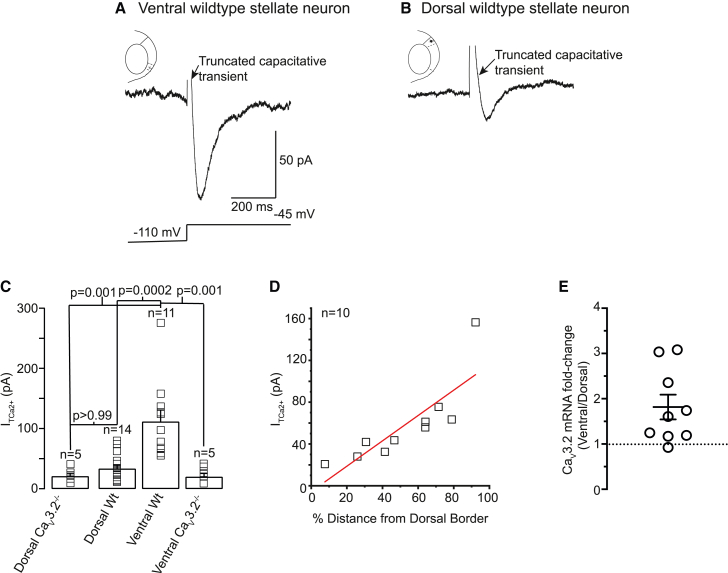
Ca_V_3 currents are present at significantly higher densities in ventral than dorsal WT neurons (A and B) Typical Ca_V_3 Ca^2+^ currents produced at the near-maximal potential of −45 mV in ventral (A) and dorsal (B) WT stellate neurons when the protocol illustrated in Figure S5A was applied. The scale associated with (A) applies to (B). (C) Average ± SEM (filled squares) and individual (open squares) T-type Ca^2+^ current amplitudes obtained in 14 dorsal WT, 11 ventral WT, 5 dorsal Ca_V_3.2^−/−^, and 5 ventral Ca_V_3.2^−/−^ neurons. Statistical significance was determined using a two-way ANOVA corrected for multiple comparisons using a Bonferroni constant. (D) Individual T-type Ca^2+^ current amplitudes recorded from stellate neurons at different locations along the dorsal-ventral border. Linear regression analysis was carried out to obtain a Pearson’s correlation coefficient. (E) Graph depicting the fold change of Ca_V_3.2 mRNA in ventral, relative to dorsal, mEC. Individual data (open circles) from nine mice and mean ± SEM are shown (p = 0.0173, one sample two-tailed test, compared with null hypothesis; dotted line shows no change).

Since T-type Ca^2+^ currents primarily affected ventral wild-type neuron activity only, we investigated whether T-type Ca_V_3.2 Ca^2+^ current amplitudes were greater in ventral versus dorsal neurons. T-type Ca^2+^ currents were recorded using whole-cell voltage-clamp conditions and the protocols shown in Figure S5 (see STAR Methods). The peak T-type Ca^2+^ current was three times larger in ventral wild-type neurons compared with their dorsal counterparts (Figure 4). The peak T-type Ca^2+^ current amplitude in dorsal wild-type neurons was similar to that in dorsal and ventral Ca_V_3.2^−/−^ neurons, implying that the dorsal wild-type neuron Ca_V_3.2 Ca^2+^ current density is minimal. As small T-type Ca^2+^ currents were present in dorsal and ventral Ca_V_3.2^−/−^ neurons, this indicated that Ca_V_3.1/Ca_V_3.3 subunits might be expressed in these neurons too. These results robustly support the notion that T-type Ca_V_3.2 Ca^2+^ currents are predominantly located in ventral wild-type neurons.

We examined whether T-type Ca^2+^ current amplitudes vary in a location-dependent manner along the dorsal-ventral axis. T-type Ca^2+^ current amplitudes in stellate neurons situated at different positions within the dorsal-ventral axis became larger from dorsal to ventral mEC regions in a distance-dependent manner (Pearson’s correlation coefficient = 0.83; Figure 4D), substantiating our notion that T-type Ca^2+^ current amplitudes increase in mEC stellate neurons along the dorsal-ventral axis.

We also assessed the T-type Ca^2+^ current biophysical properties in stellate neurons, particularly as the currents had voltage-dependent effects on R_N_ (Figures 2, 3, and S5; Tables S1 and S2). The small T-type Ca^2+^ currents in dorsal neurons precluded us from investigating their biophysical characteristics. In ventral wild-type neurons, T-type Ca^2+^ currents had an average rise time constant of 11.9 ± 2.08 ms (n = 7) and decay time constant of 77.5 ± 10.91 ms (n = 7) at −45 mV (Figure S5). The activation and inactivation curves had half-maximal voltages (V_1/2_) of −53.9 ± 1.9 mV and −79.7 ± 1.3 mV respectively (Figure S5), consistent with those reported previously.^22^ The slope (k) values of the activation and inactivation curves were 7.9 ± 1.0 mV^−1^ and 7.5 ± 0.9 mV^−1^ respectively (Figure S6C). These values agree with those of expressed Ca_V_3.2 Ca^2+^ channels in heterologous systems,^27^ providing additional evidence that Ca_V_3.2 channels predominantly underlie the T-type Ca^2+^ current in ventral wild-type neurons.

To test whether Ca_V_3.2 expression differs between dorsal and ventral wild-type neurons, we performed quantitative reverse-transcriptase (RT) PCR using micro-dissected adult mEC layer II–III dorsal or ventral tissue (see STAR Methods). The mRNA level for Ca_V_3.2 was greater in ventral compared with dorsal mEC in eight out of wild-type mice examined. Using ΔΔC_T_ values, the fold increase in ventral relative to dorsal tissue was 1.82 ± 0.27 (p = 0.0173; Figure 4E). These results further support the notion that ventral stellate neurons have a greater Ca_V_3.2-mediated T-type Ca^2+^ current than dorsal stellate neurons.

### Computational modeling indicates that T-type Ca^2+^ currents act in concert with subthreshold Na^+^ currents to enhance ventral wild-type stellate neuron R_N_

**Figure 5:**
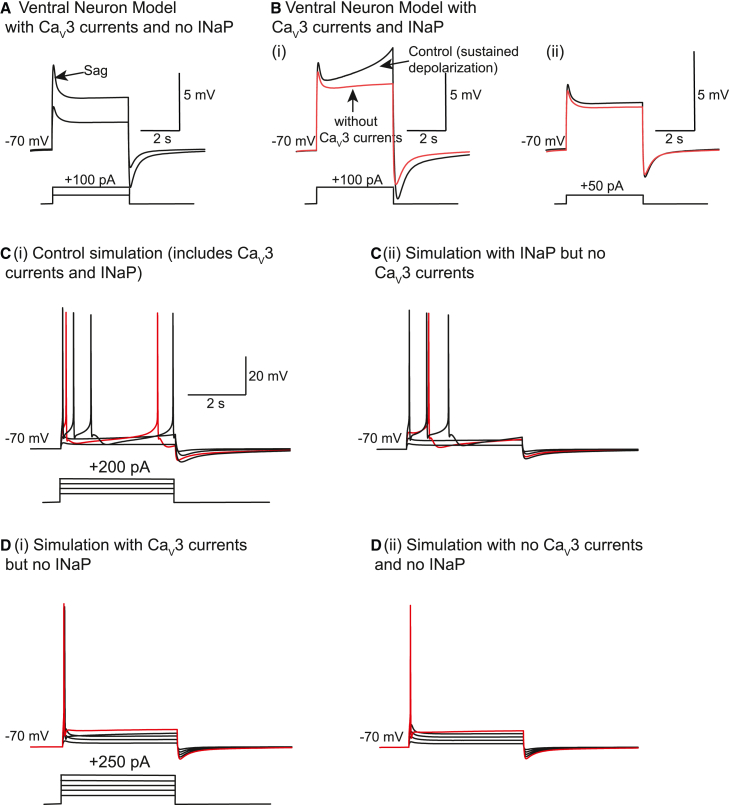
Computational modeling indicates that Ca_V_3 currents act together with subthreshold Na^+^ currents to enhance ventral stellate neuron R_N_ and excitability (A) Simulations obtained when HCN, K^+^, the transient Na^+^, and T-type Ca_V_3 Ca^2+^ currents were only included. (B) Traces acquired with +100 pA (i) and +50 pA (ii) steps when the model incorporated persistent Na^+^ currents (INaP) in addition to the currents in (A). Simulations in red were obtained when Ca_V_3 currents were removed. (C) Simulations attained in the presence (i) and absence (ii) of T-type Ca^2+^ currents when depolarizing steps were applied as shown in the schematic in (i). The simulation obtained with +150 pA current injection is highlighted in red. (D) Simulations obtained in the absence of INaP (i) and INaP together with Ca_V_3 currents (ii) when depolarizing steps were applied as shown in the schematic in (i). The simulation obtained with +250 pA current injection is highlighted in red. The scale shown in (C(i)) applies to all simulations in (C) and (D).

As T-type Ca_V_3.2 Ca^2+^ currents are fast inactivating in stellate neurons, how might these currents cause long-lasting depolarizations with subthreshold depolarizing stimuli in ventral neurons? To understand this, we generated a computational model of a ventral stellate neuron that incorporated known ion channel conductances as well as our experimental T-type Ca^2+^ current kinetics and density measurements obtained from the soma (Table S4; STAR Methods). A caveat of this approach is that the ion channel conductances and their distribution in our model neuron might not fully represent the native conditions. Nonetheless, this approach is informative as a first step in understanding how T-type Ca^2+^ channels influence ventral neuron R_N_ and action potential firing. Using this model, subthreshold depolarizing pulses resulted in a sag but no sustained depolarization (Figure 5A). The R_N_ measured with +50 pA and +100 pA steps was 40 MΩ and 45 MΩ, which was substantially lower than that obtained experimentally (Figure 2; Table S1). This suggests T-type Ca^2+^ currents alone were unlikely to sustain a depolarization in ventral neurons and indicate that, for this purpose, they need to act in concert with other subthreshold-activated voltage-gated currents.

Ventral neurons possess axo-somatic subthreshold-activated persistent Na^+^ currents.^28^^,^^29^^,^^30^ We asked whether this conductance together with the somatic T-type Ca^2+^ conductance would reproduce the characteristic sustained depolarization with subthreshold current pulses in ventral neurons in our model (Figures 2, 3, and S3). Including the persistent Na^+^ conductance in soma and axons together with a somatic T-type Ca^2+^ conductance resulted in a prolonged depolarization with +100 pA steps (Figure 5B). The R_N_ values measured under these conditions using +50 pA and +100 pA steps were 60 MΩ and 100 MΩ respectively and concur with our experimental findings (Figure 2).

We next tested whether T-type Ca^2+^ conductance removal altered R_N_ under these conditions. T-type Ca^2+^ conductance deletion no longer produced a sag and no sustained depolarization with +100 pA steps (Figure 5B(ii)). R_N_ values measured with +50 pA and +100 pA steps were reduced to 45 MΩ and 55 MΩ respectively. These values were similar to those obtained when the persistent Na^+^ conductance was not incorporated in the model. Indeed, with no T-type Ca^2+^ conductance, further removal of the persistent Na^+^ conductance had little effect on R_N_. Hence, the model suggests that T-type Ca^2+^ currents activate persistent Na^+^ currents to exert their effects in ventral neurons.

Our experimental results also indicate that T-type Ca^2+^ currents enhance spike firing by elevating R_N_ at positive voltages. In agreement with our experimental findings (Figures 1 and S3), T-type Ca^2+^ current removal reduced spike numbers elicited by depolarizing pulses ≥+150 pA compared with controls (Figure 5C), providing additional evidence that T-type Ca^2+^ currents enhance ventral neuron action potential firing rates.

As the model predicts that T-type Ca^2+^ conductance requires, at least, a persistent Na^+^ conductance to be present to have sustained effects on R_N_, we investigated how T-type Ca^2+^ conductance would affect action potential firing when there was no persistent Na^+^ conductance. Persistent Na^+^ conductance removal significantly reduced action potential firing (Figure 5D(i)), consistent with the reduced R_N_ under these conditions. Subsequent abolition of the T-type Ca^2+^ conductance had little further effect on spike numbers under these conditions (Figure 5D(ii)), indicating that T-type Ca^2+^ currents and persistent Na^+^ currents act in conjunction to modulate ventral neuron firing.

### T-type Ca^2+^ currents and persistent Na^+^ currents together enhance ventral wild-type neuron excitability

**Figure 6:**
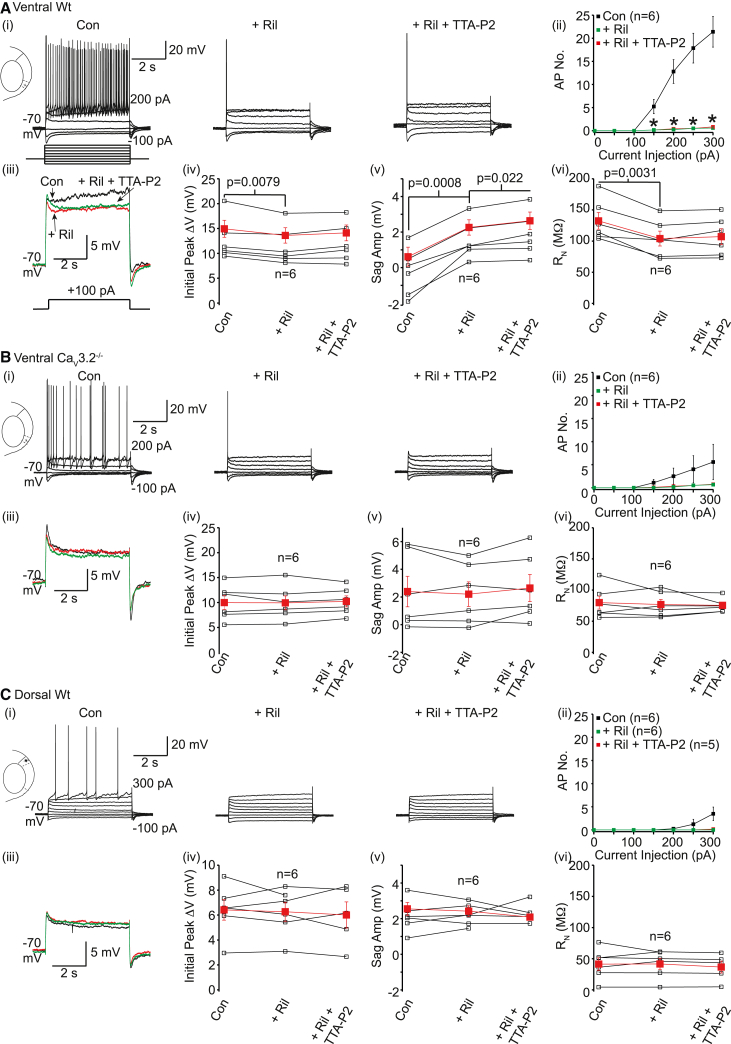
Subthreshold Na^+^ current inhibition prevents the effects of T-type Ca^2+^ channel inhibitors on ventral, and not dorsal, WT intrinsic excitability (A–C) Representative recordings from (A(i)) ventral WT, (B(i)) ventral Ca_V_3.2^−/−^, and (C(i)) dorsal WT stellate neurons in the absence or presence of riluzole (Ril) and subsequent co-application of riluzole and TTA-P2 when the protocol shown was applied. The minimum and maximum current injections are indicated next to the first trace. The scale associated with the first trace applies to all traces within the panel. (A(ii), B(ii), and C(ii)) Average ±SEM action potential numbers in the absence (con), presence of riluzole and subsequent co-application of riluzole and TTA-P2 obtained from six ventral WT, six ventral Ca_V_3.2^−/−^, and six dorsal WT neurons respectively. Paired t tests were performed to obtain significance (p) values. Asterisks indicate significance at p < 0.05 (exact p values are stated in Table S7). (A(iii), (Biii), and C(iii)) Superimposed typical traces obtained when a +100 pA step was applied under control conditions (black), with riluzole (red), and following co-application of riluzole and TTA-P2 (green). (A(iv), A(v), A(vi), B(iv), B(v), B(vi), C(iv), C(v), and C(vi)) Individual (open squares) and mean ± SEM (filled red square) initial peak voltage change (ΔV), sag amplitude (amp), and R_N_ values under control conditions, following application of riluzole and after subsequent co-application of riluzole and TTA-P2 6 from six ventral WT, six ventral Ca_V_3.2^−/−^, and six dorsal WT neurons respectively. Significance (p) values indicated on graphs were determined using paired t tests.

To experimentally test the notion that T-type Ca^2+^ currents act in concert with persistent Na^+^ currents to cause sustained depolarization with subthreshold pulses and increase ventral wild-type neuron activity, we applied the persistent Na^+^ channel inhibitor, riluzole (10 μM), followed by co-application of riluzole and TTA-P2. Riluzole had little effect on the RMP or R_N_ and sag amplitude due to hyperpolarizing pulses in wild-type and Ca_V_3.2^−/−^ neurons (Table S5). Riluzole, though, significantly reduced the number of action potentials generated with depolarizing steps in wild-type neurons (Figure 6A(i)). Subsequent co-application of riluzole and TTA-P2 had little further effect on spike firing in these neurons (Figures 6A(i) and 6A(ii)). Riluzole reduced wild-type R_N_ by 22.8% ± 5.4% (n = 6; Table S5) and 21.7% ± 4.3% (n = 6; Figure 6A) when measured with +50 pA and +100 pA steps respectively. R_N_ was decreased to a similar extent with the Na^+^ channel inhibitor, TTX (1 μM; Figure S6; Table S6). Co-application of riluzole and TTA-P2 or TTX and TTA-P2 then did not further affect R_N_ (Figure 6A; Table S5), suggesting that T-type Ca^2+^ currents are unable to modulate R_N_ in the absence of Na^+^ currents in wild-type neurons (Figure S6; Table S6). Application of riluzole (Figure 6A(iv); Table S5) or TTX (Figure S6; Table S6) depressed the initial peak voltage change induced by depolarizing pulses in wild-type neurons. This was not further changed by co-application of riluzole/TTX and TTA-P2 (Figure 6A(iv) and S6; Tables S5 and S6). Riluzole or TTX application enhanced the sag amplitude with +100 pA steps too (Figures 6A and S6). The sag produced using +100 pA steps was further increased by co-application of riluzole and TTA-P2 (Figure 6A(v); Table S5), but not TTX and TTA-P2 (Figure S6C). Nonetheless, since both TTX and riluzole reduced R_N_ that was subsequently not further modified by TTA-P2, and as riluzole is a persistent Na^+^ current inhibitor,^28^ these results suggest that T-type Ca^2+^ currents act in concert with persistent Na^+^ currents to enhance R_N_.

Our model (Figure 5) also implied that, in the absence of T-type Ca^2+^ currents, persistent Na^+^ currents have little effect on R_N_, suggesting a co-dependence of T-type Ca^2+^ and persistent Na^+^ currents in modifying ventral neuron excitability. To test this, we applied riluzole onto ventral Ca_V_3.2^−/−^ neurons (Figure 6B; Table S5). Riluzole had little effect on action potential firing in these neurons (Figures 6B(i) and 6B(ii)). Neither riluzole nor subsequent co-application of riluzole and TTA-P2 had any effect on the initial peak voltage change, sag amplitude, or R_N_ measured with +50 pA or +100 pA steps in Ca_V_3.2^−/−^ neurons (Figure 6B; Table S5). The initial peak voltage depolarizations for these steps were similar between wild-type and Ca_V_3.2^−/−^ neurons (Figures 6A and 6B; Table S5), even though R_N_ measured with +50 pA or +100 pA steps was significantly reduced in Ca_V_3.2^−/−^ neurons compared with wild-types (Ca_V_3.2^−/−^ R_N_ for +50 pA and +100 pA = 65.07 ± 8.3 MΩ [n = 6] and 79.78 ± 10.4 MΩ [n = 6] respectively; wild-type R_N_ for +50 pA and +100 pA = 100.00 ± 10.9 MΩ [n = 6, p = 0.025] and 132.44 ± 13.4 MΩ [n = 6, p = 0.001] respectively). These findings suggest that larger depolarizing stimuli might be required to evoke persistent Na^+^ currents in Ca_V_3.2^−/−^ neurons. Since the initial peak voltage change with +150 pA step (15.13 ± 2.8 mV, n = 6) was greater than for +100 pA step (9.99 ± 1.4 mV, p = 0.02, paired t test), we tested the effects of riluzole on +150 pA steps. While the initial peak voltage and sag amplitudes were unaffected by riluzole, R_N_ was reduced from 260.0 ± 49.2 MΩ (n = 6) under control conditions to 216.27 ± 31.4 (n = 6; p = 0.036, paired t test) with riluzole. Hence, ventral Ca_V_3.2^−/−^ neurons possess persistent Na^+^ currents but a greater depolarizing current is required to activate these currents. These findings support the notion that T-type Ca_V_3.2 Ca^2+^ currents in ventral wild-type neurons facilitate the activation of persistent Na^+^ currents.

### Persistent Na^+^ current activation requires larger depolarizing currents in dorsal wild-type neurons compared with ventral wild-type neurons

Dorsal wild-type neurons have larger persistent Na^+^ currents than ventral neurons, although it is unknown if their biophysical properties differ along the dorsal-ventral axis.^28^ As dorsal neurons have at least 3-fold lower T-type Ca^2+^ current amplitudes than ventral neurons (Figure 4C), we asked how persistent Na^+^ current inhibition affects their excitability. 10 μM riluzole or subsequent co-application of riluzole and TTA-P2 had little effect on the RMP, firing rates, or R_N_ and sag amplitude due to hyperpolarizing or subthreshold depolarizing (+50 pA and +100 pA) current pulses in dorsal neurons (Figure 6C; Table S5). The depolarization induced by these subthreshold steps in dorsal neurons is less than in ventral neurons as the initial peak voltage change due to +50 pA and +100 pA steps was significantly smaller in dorsal neurons compared with ventral neurons (Figure 6C; Tables S1 and S5). The initial peak voltage change, though, with +150 pA (9.10 ± 1.04 mV, n = 6) and +200 pA (11.28 ± 1.21 mV, n = 6) steps in dorsal neurons was similar to that produced by +100 pA steps (14.94 ± 1.7 mV, n = 6) in ventral neurons. Since dorsal wild-type neurons rarely fire action potentials with current injections up to +200 pA (Figure 1), we investigated whether riluzole affected R_N_ when these larger steps were applied. While riluzole and subsequent co-application of riluzole with TTA-P2 had little effect on R_N_, initial peak voltage change, or sag amplitude measured using +150 pA steps (Figure S7A), riluzole reduced R_N_ measured with +200 pA by 11.5% ± 2.7% (n = 6) in these neurons and enhanced the sag amplitude (Figure S7B). It is worth noting that riluzole had a much smaller effect on R_N_ measured with +200 pA in dorsal neurons than in ventral neurons when R_N_ was measured with smaller current pulses. Riluzole also inhibited subthreshold oscillations in these neurons generated by >+200 pA depolarizing steps, consistent with previous reports.^31^ Altogether these findings suggest that activation of persistent Na^+^ currents requires larger depolarizing currents in dorsal wild-type neurons compared with ventral neurons. This is likely to be due to dorsal neurons having higher densities of K^+^ and HCN currents and a low density of T-type Ca_V_3.2 Ca^2+^ currents compared with ventral neurons.

### Ca_V_3.2 channels enhance summation of EPSPs and augment EPSP-spike coupling in ventral wild-type mEC stellate neurons

**Figure 7:**
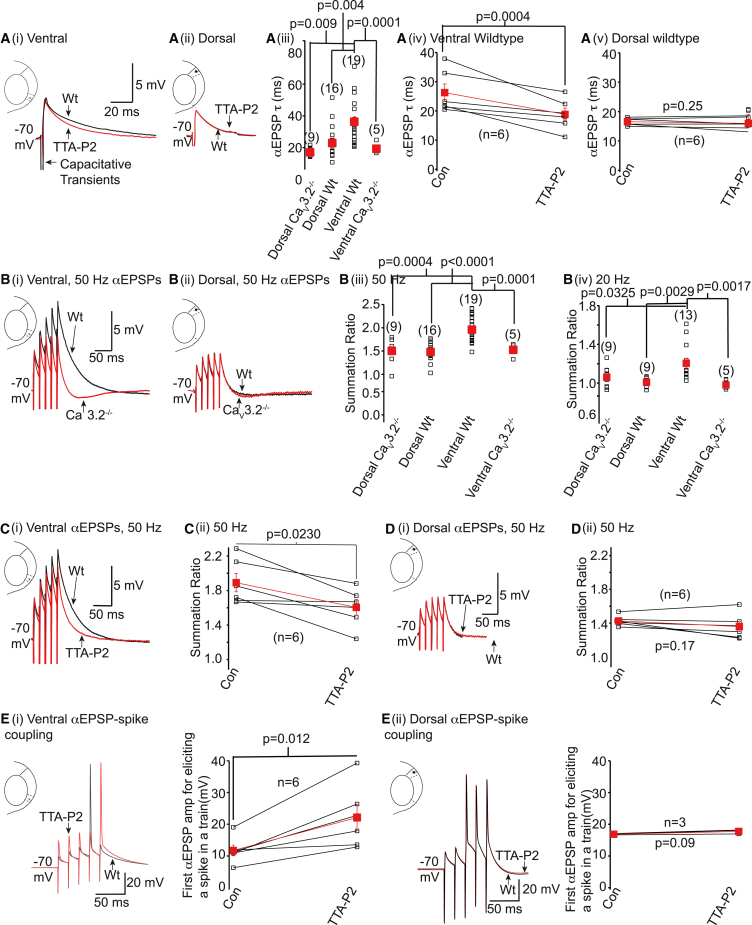
Ca_V_3.2 Ca^2+^ currents regulate αEPSP decay time constants and summation in ventral WT stellate neurons alone (A(i), A(ii)) Example single αEPSP recordings under control conditions and in the presence of TTA-P2. The scale shown in (A(i)) applies to (A(ii)). (A(iii)) Individual (black squares) and mean ± SEM (red squares) αEPSP decay time constants (τ) from 9 dorsal Ca_V_3.2^−/−^, 16 dorsal WT, 19 ventral WT, and 5 ventral Ca_V_3.2^−/−^ neurons. To determine significance, data from all groups were compared using a two-way ANOVA with Fisher’s LSD *post hoc* test. (A(iv) and A(v)) Individual (open squares) and average ±SEM (filled squares) single αEPSP decay time constant (τ) values under control conditions and following TTA-P2 obtained from 6 ventral and 6 dorsal WT neurons, respectively. Significance (p) values were determined using paired t tests. (B(i), B(ii), C(i), and D(i)) Example recordings of a 50-Hz train of five αEPSPs in WT, Ca_V_3.2^−/−^ neurons, or in the presence of TTA-P2. (B(iii) and B(iv)) Summation ratios obtained from 9 dorsal Ca_V_3.2^−/−^, 16 dorsal WT, 19 ventral WT, and 5 ventral Ca_V_3.2^−/−^ neurons when trains of five αEPSPs at 50 Hz and 20 Hz were applied respectively. Data from all groups were compared using a two-way ANOVA with Fisher’s LSD *post hoc* test to ascertain the p values. (C(ii) and D(ii)) Summation ratio values obtained from 6 ventral and 6 dorsal WT neurons when trains of 50 Hz αEPSPs were applied in the absence (con) and presence of TTA-P2. Paired t tests were used to obtain p values. (E(i) and E(ii)) Representative traces showing αEPSP-spike coupling under control conditions (black) and after TTA-P2 (red). The graphs on the right show the first αEPSP amplitude values in a 50-Hz train of five αEPSPs that produced a spike in 6 ventral and 3 dorsal WT neurons. Paired t tests were performed to obtain p values.

*In vivo*, neuronal activity is strongly influenced by EPSP generation and integration. Thus, we investigated whether Ca_V_3.2 Ca^2+^ currents influenced EPSP shapes in stellate neurons and thereby modulate their activity. Ca_V_3.2 Ca^2+^ channels are present presynaptically in the mEC and influence synaptic release under certain conditions.^23^ To avoid the additional complexity caused by possible differences in endogenous synaptic release onto neurons, we generated EPSPs by injecting current waveforms using a defined α function (αEPSPs; see STAR Methods). The rise time of αEPSPs was set to be 1 ms under control conditions. The current injection was adjusted so that the αEPSP amplitude was between 1 and 5 mV, with no difference in the αEPSP amplitudes between all groups (ventral wild-type, ventral Ca_V_3.2^−/−^, dorsal wild-type, and dorsal Ca_V_3.2^−/−^ αEPSP amplitudes were 3.77 ± 0.35 mV [n = 19], 2.79 ± 0.70 mV [n = 5], 3.55 ± 0.35 mV [n = 16], and 2.38 ± 0.34 mV [n = 9] respectively). The αEPSP decay time constant, though, was significantly greater in ventral wild-type neurons compared with the other groups (Figure 7A). Further, TTA-P2 decreased αEPSP decay time constants in ventral, but not dorsal, wild-type neurons (Figure 7A). Certainly, 50- or 20-Hz trains of αEPSPs summed significantly more in ventral wild-type neurons compared with ventral Ca_V_3.2^−/−^ neurons or dorsal neurons (Figure 7B). Consistent with these findings, TTA-P2 applied onto ventral, but not dorsal, wild-type stellate neurons reduced the summation of a 50-Hz train of five αEPSPs (Figures 7C and 7D). These findings suggest that Ca_V_3.2 Ca^2+^ channels influence EPSP integration in ventral, but not dorsal, neurons.

We also tested whether αEPSP-spike coupling differed between these neurons by eliciting five αEPSPs at gamma frequency (50 Hz) as synaptic input to these neurons has been suggested to cause intrinsic gamma oscillations in these neurons, which may resemble their activity during spatial exploration.^32^ In ventral wild-type neurons, a train of five αEPSPs at 50 Hz summated sufficiently to produce a single spike with the fourth or fifth αEPSP when the first αEPSP amplitude was 11.74 ± 1.68 mV (n = 6; Figure 7E(i)). The summation ratio was 2.00 ± 0.16 (n = 6; i.e., similar to when the first αEPSP amplitude was 1–5 mV (Figure 7B(iii)). TTA-P2 reduced the summation ratio to 1.49 ± 0.09 (n = 6, p = 0.022). Consequently, the first αEPSP amplitude of the 50-Hz train required to elicit a single spike was 22.12 ± 4.05 mV (n = 6, p = 0.013). In dorsal wild-type neurons, the average first αEPSP amplitude in a train of five αEPSPs at 50 Hz required to elicit spikes was 21.71 ± 3.55 mV (n = 7), i.e., significantly (p = 0.033) greater than in ventral neurons. Moreover, the summation ratio (1.07 ± 0.14, n = 7) of these trains in dorsal neurons was substantially (p = 0.001) less than that in ventral neurons. As expected, the summation ratios of five αEPSPs at 50 Hz in dorsal neurons were similar before (0.81 ± 0.35, n = 3) and after TTA-P2 application (1.21 ± 0.01, n = 3, p = 0.18). TTA-P2, therefore, did not affect the first αEPSP amplitude required to elicit EPSP-spike coupling in dorsal neurons (Figure 7E(ii)). In five out of seven dorsal neurons, though, five αEPSPs at 50 Hz generated two or three consecutive spikes (see Figure 7E(ii)) and not single spikes, analogous to that observed by Bant et al.^28^ This supports the notion that the higher density of Na^+^ currents in these neurons enables multiple spikes to be elicited with trains of large-amplitude EPSPs.^28^

A further question is whether the increase in αEPSP-spike coupling by T-type Ca^2+^ channels in ventral wild-type neurons is frequency dependent. Given that five αEPSPs of 1–5 mV summate less at 20 Hz (1.2 ± 0.05, n = 13) than 50 Hz (2.0 ± 0.07, n = 19, p < 0.01, Figure 7B), we tested whether TTA-P2 affected αEPSP-spike coupling elicited at 20 Hz. The first αEPSP amplitude for inducing spikes in a train of five αEPSPs at 20 Hz was approximately double (20.1 ± 3.8 mV, n = 6) that at 50 Hz, with αEPSP summation at 20 Hz (1.3 ± 0.2, n = 6) being very similar to that produced with αEPSPs of 1–5 mV (Figure 7B(iv)). TTA-P2 application reduced αEPSP summation (1.05 ± 0.1, n = 6, p = 0.046) so that the first αEPSP amplitude for αEPSP-spike coupling was significantly greater (32.8 ± 4.7 mV, n = 6, p = 0.004). These findings suggest that T-type Ca^2+^ channels influence αEPSP spike coupling even with lower-frequency αEPSP trains. Hence, T-type Ca^2+^ currents play a critical role in modulating ventral stellate neuron synaptic and intrinsic excitability.

## Discussion

There is increasing evidence that the mEC supports a variety of functions by having within-cell-type heterogeneity to allow implementation of discrete computations.^1^ Certainly, ventral mEC layer II stellate neuron action potential firing rates are significantly higher compared with their dorsal counterparts, as ventral neurons have a greater R_N_ than dorsal neurons. Here, we show that this greater R_N_ is, at least partly, due to a 3-fold greater density of T-type Ca^2+^ currents in ventral neurons compared with dorsal neurons. These currents act in concert with persistent Na^+^ currents in ventral, but not dorsal, neurons to augment R_N_ and elevate their firing frequency. T-type Ca_V_3.2 Ca^2+^ currents themselves also prolonged αEPSP decay and enhanced αEPSP summation in ventral neurons. Consequently, a smaller-amplitude first αEPSP caused αEPSP-spike coupling in ventral neurons compared with dorsal neurons, suggesting that T-type Ca^2+^ currents in ventral neurons promote spike-timing synaptic plasticity. Thus, our findings imply that the greater T-type Ca_V_3.2 Ca^2+^ subunit expression in ventral versus dorsal neurons may be used as a tool for discerning how ventral neurons influence mEC neural network activity and associated diverse functions.

### T-type Ca^2+^ channels facilitate persistent Na^+^ current activation in mEC ventral stellate neurons

T-type Ca^2+^ currents exerted their effects in ventral neurons by activating persistent Na^+^ currents. Large depolarizing stimuli activated T-type Ca^2+^ currents to cause the membrane to remain depolarized longer and thereby mask the sag due to HCN channel de-activation. Our results suggest that this triggered persistent Na^+^ currents to produce a sustained membrane potential depolarization. Consequently, ventral neuron R_N_ was enhanced and action potential firing increased. Certainly, if persistent Na^+^ currents were inhibited, T-type Ca^2+^ currents were unable to affect ventral neuron R_N_. Further, in the absence of Ca_V_3.2 Ca^2+^ currents, greater depolarizing pulses were required for persistent Na^+^ currents to be stimulated in ventral neurons and persistent Na^+^ current inhibition had little effect on action potential firing. Thus, our findings point to a distinct mechanism by which T-type Ca^2+^ channels and persistent Na^+^ currents act in concert to enhance ventral wild-type neuron firing.

The interaction between T-type Ca^2+^ channels and persistent Na^+^ channels is likely to be voltage dependent as this effect was reproducible in a simple ventral neuron computational model in which the persistent Na^+^ current biophysical characteristics were not altered following T-type Ca^2+^ current activation. Interestingly, though, in hippocampal and cerebellar neurons, Ca^2+^ entry via Ca^2+^ channels rapidly phosphorylates Na^+^ channels to enhance the persistent Na^+^ current.^30^^,^^33^^,^^34^^,^^35^ Moreover, Ca^2+^∕calmodulin-dependent kinase (CaMKII) phosphorylates Na_V_1.2 channels, which predominantly underlies transient Na^+^ currents, to generate a persistent Na^+^ current in heterologous systems.^36^ Thus, while we cannot exclude the possibility that Ca^2+^ entry via T-type Ca^2+^ channels may have altered Na^+^ channel biophysical properties in ventral neurons, this is unlikely as T-type Ca^2+^ currents were not active at RMP. Indeed, unlike in thalamic neurons,^25^ there is only a small window T-type Ca^2+^ current in ventral neurons (Figure S6C), which did not alter the RMP, the initial voltage jump caused by depolarizing stimuli, or the ability to generate intrinsic oscillations. However, the window T-type Ca^2+^ current might be enhanced under particular conditions as in mEC synaptic terminals^23^ and may then influence ventral neuron activity.

In many neurons, the rebound potential following a hyperpolarizing pulse activates T-type Ca^2+^ channels and facilitates action potential generation.^17^ While there was a rebound potential following a hyperpolarizing pulse in ventral wild-type neurons, the amplitude of this was similar irrespective of Ca_V_3.2 Ca^2+^ currents and no action potentials were generated with rebound potentials under control conditions. Hence, T-type Ca^2+^ currents influence activity in an unusual manner in stellate neurons compared with other neurons such as thalamic neurons.

As our electrophysiological recordings were all performed at the soma, our work strongly suggests that somatic T-type Ca^2+^ channels interact with axo-somatic persistent Na^+^ currents to influence ventral stellate wild-type neuron R_N_ and excitability. This interaction, though, may also be present in other neuronal compartments such as axons and dendrites, as both T-type Ca^2+^ and persistent Na^+^ channels are localized in these compartments in other neurons, including those within the mEC.^17^^,^^19^^,^^23^^,^^34^^,^^37^^,^^38^^,^^39^^,^^40^^,^^41^ The co-existence of T-type Ca^2+^ and persistent Na^+^ currents in these compartments might serve to facilitate EPSP integration and/or action potential generation as in the soma.

### T-type Ca^2+^ currents and ventral mEC circuit activity

*In vivo*, mEC layer II stellate neuron firing activity is associated with grid cell patterns.^1^^,^^41^^,^^42^ Interestingly, the grid cell firing fields get larger along the dorsal-ventral mEC axis,^1^ corresponding to the increase in mEC layer II stellate neuron firing along the dorsal-ventral axis.^14^ As ventral mEC neuron axons project to ventral hippocampus, changes in ventral mEC grid spacing are predicted to alter ventral place cell activity.^4^ Certainly, selectively reducing HCN1 activity in mEC stellate neurons, which enhances their firing rate, expanded grid cell size and spacing in mEC along the dorsal-ventral axis and decreased place cell stability in hippocampal CA1 area.^4^ By analogy, as T-type Ca_V_3.2 Ca^2+^ channels predominantly increase ventral mEC stellate neuron firing, their presence in these neurons might contribute to the increased grid cell size and spacing in ventral mEC compared with dorsal mEC. This in turn may affect place cell stability in the hippocampus, which may influence the brain’s ability to code information related to spatial awareness. It should be noted, though, that grid cells are produced by coherent neural network activity and their patterns are strongly influenced by excitatory and inhibitory synaptic connectivity.^1^ As T-type Ca_V_3.2 Ca^2+^ currents are present in other cell types within the mEC and are also present presynaptically where they influence synaptic transmission under certain conditions,^23^ the overall impact of modified expression of these channels on grid cell activity will be complex. Nonetheless, targeted alteration of Ca_V_3.2 subunit expression in ventral stellate neurons alone may be beneficial for testing the impact of selectively modulating their activity on grid cell size and spacing.

In addition to a physiological role in spatial memory formation, mEC neurons may significantly contribute to seizure generation and propagation during temporal lobe epilepsy.^43^ In animal models, mEC stellate neurons are hyperexcitable following initiation of seizures, which has been, in part, attributed to increased expression of T-type Ca_V_3.1 Ca^2+^ channel subunits.^44^ Persistent Na^+^ currents have also been suggested to be enhanced in these neurons post seizures in rodents.^45^ While it is unknown if these augmentations in T-type Ca^2+^ currents and persistent Na^+^ currents occur across the dorsal-ventral axis in these neurons after seizure activity, our findings support the notion that that these currents will act in concert to substantially enhance stellate neuron excitability following seizures. Given that stellate neuron axons project to the hippocampus,^3^ their augmented excitability mediated by T-type Ca^2+^ and persistent Na^+^ currents may serve to facilitate hippocampal neuron hyperexcitability following seizures.

In summary, we have shown that the enhanced intrinsic excitability of ventral mEC stellate neurons compared with dorsal stellate neurons is at least partly due to the high expression of T-type Ca_V_3.2 Ca^2+^ currents in ventral neurons. These currents act in concert with persistent Na^+^ currents to elevate ventral stellate neuron activity. As ventral and dorsal mEC layer II circuit connections are distinctive,^2^^,^^46^^,^^47^^,^^48^ the increased T-type Ca^2+^ currents in ventral mEC layer II neurons compared with dorsal mEC layer II neurons may contribute to diversely influencing neural circuit activity such as grid cells and functions such as spatial memory.

### Limitations of the study

While we found that T-type Ca^2+^ currents and persistent Na^+^ currents act in concert at the soma, it is possible that this interaction may occur in other neuronal compartments, such as axons and dendrites. This needs to be further assessed. Moreover, the consequences of the high T-type Ca^2+^ current in ventral mEC stellate neurons on grid cell size and spacing in this region requires investigation. Finally, it will be fascinating to ascertain whether our findings in mEC stellate neurons might also be applicable to other brain regions where there is known within-cell-type heterogeneity.^2^

## STAR★Methods

### Key resources table

**Table:** 

REAGENT or RESOURCE	SOURCE	IDENTIFIER
**Antibodies**		

Alexa Fluor 488 streptavidin conjugate antibody	ThermoFisher Scientific	S11223

**Chemicals, peptides, and recombinant proteins**		

Ketamine/Xylazine mixture	Sigma-Aldrich UK	K113
Choline Chloride	Sigma-Aldrich UK	C7527
Sodium Chloride	Fisher Scientific UK	10092740
Potassium Chloride	Fisher Scientific UK	10730492
Sodium phosphate	Sigma-Aldrich UK	S9638
Sodium bicarbonate	Sigma-Aldrich UK	S6014
Calcium chloride 1M solution	Sigma-Aldrich UK	21115
Magnesium chloride hexahydrate	Sigma-Aldrich UK	M2393
D-glucose	VWR UK	24379.294
Potassium Methyl Sulfate	Fisher Scientific UK	0342333
HEPES	Sigma-Aldrich UK	H4034
EGTA	Sigma-Aldrich UK	324626
Na_2_ATP	Sigma-Aldrich UK	A7699
Tris-GTP	Sigma-Aldrich UK	G1009
Tris-phosphocreatinine	Sigma-Aldrich UK	P1937
Neurobiotin	Vector Labs UK	SP110
CNQX disodium salt	Abcam UK	ab120044
DL-AP5 sodium salt	Abcam UK	ab120271
Bicuculline	Abcam UK	ab120107
CGP 55845	Tocris UK	1248/10
Tetrodotoxin citrate	Abcam UK	ab120055
Cesium chloride	Sigma-Aldrich UK	C3139
Tetraethylammonium chloride	Tochris	3068
4-aminopyradine	Sigma-Aldrich UK	A78403
Nifedipine	Sigma-Aldrich UK	N7634
ω-agatoxin IVA	Tebu Bio	11AGA001-00100
ω-conotoxin GVIA	Tebu Bio	08CON001-00500
TTA-P2	Gift from Merck Research Laboratories	N/A
NiCl_2_	Sigma-Aldrich UK	N6136
Riluzole	Sigma-Aldrich UK	R116
Paraformaldehyde	Electron Microscopy Services	30525-89-4
Phosphate Buffer Solution Tablets	Fisher Scientific UK	12821680

**Critical commercial assays**		

RNeasy Lipid Tissue Kit	Qiagen	74004
High capacity RNA-to-cDNA kit	Applied Biosciences	4387406
TaqMan Gene Expression Master Mix	ThermoFisher Scientific	4369016

**Experimental models: Organisms/strains**		

Ca_V_3.2 mouse heterozygotes	Prof. K. Campbell (University of Iowa)	N/A

**Software and algorithms**		

pClamp 10.4	Molecular Devices	N/A
Model DB	Yale University	N/A
Ventral Cell Model and Simulation Files	ModelDB	https://senselab.med.yale.edu/modeldb/266797

### Resource availability

#### Lead contact

Further information and requests for resources and reagents should be directed to and fulfilled by the lead contact, Mala shah (mala.shah@ucl.ac.uk).

#### Materials availability

The study did not generate new unique reagents.

### Experimental model and subject details

All animal experiments were approved by the UCL animal welfare and ethics review body (AWERB) and were performed with approved UK Home Office personal and project licenses. All animals were housed under 12 h dark/light cycles and were provided with *ad libitum* food and water. Ca_V_3.2 heterozygotes (Ca_V_3.2^+/−^) breeding pairs were used to generate adult (5–8 week old) male and female wildtype and Ca_V_3.2 null (Ca_V_3.2^−/−^) littermates for experiments (see^23^^,^^49^ for Methods).

### Method details

#### Acute slice preparation

5-8 week old wildtype and Ca_V_3.2^−/−^ mice were terminally anesthetized using a ketamine/xylazine mixture. For initial experiments for Figures 1, 2, 3, 4, S3, and S4, the experimenter was blind to the mouse genotype until data analysis had been completed. Intra-cardiac perfusion was then carried out using an ice-cold modified artificial cerebral spinal fluid (ACSF) of the following composition (mM): 2.5 KCl, 1.25 NaH_2_PO_4_, 25 NaHCO_3_, 0.5 CaCl_2_, 7 MgCl_2_, 7 dextrose, 110 choline chloride. 300 μm thick parasagittal slices were obtained using an LT 1200S vibratome (Leica Microsystems, UK). Slices containing the mEC were identified and selected as described in ref. 24. These slices were transferred to a chamber maintained at 35°C and containing ACSF (mM): 125 NaCl, 2.5 KCl, 1.25 NaH_2_PO_4_, 25 NaHCO_3_, 2 CaCl_2_, 2 MgCl_2_, 10 dextrose, pH 7.3 maintained with 95% O_2_/5% CO_2_ mixture. Slices were stored at 35°C for 25 min, after which they were kept at room temperature for 40 min.

#### Electrophysiological recordings

For patch-clamp recordings, the slices were transferred to a submerged chamber perfused at 3–5 mL/min with ACSF supplemented with 10 μM CNQX, 50 μM DL-AP5, 10 μM bicuculline and 1 μM CGP 55845 and maintained at 32–36°C. The dorsal and ventral mEC were initially identified under low magnification (x10) using an Olympus BX5141 microscope (Microscope Service and Sales, UK). The parasubicular/dorsal mEC border could be visualized using low magnification ×10 objectives under a microscope (Olympus B×51W1) equipped with differential infrared contrast optics and was readily visible as a dark band. The ventral mEC border was estimated from position of the CA1/subicular border.^24^ Stellate neurons located within 30% of the mEC dorsal border was classified as dorsal (Figures S1 and 1A). Correspondingly, those situated within 30% of the ventral border were categorized as ventral (Figures S1 and 1B).

##### Current-clamp recordings

Whole-cell current-clamp recordings were obtained from visually-identified stellate neurons present in either dorsal or ventral regions of the slice. Patch pipettes had a resistance of 5–8 MΩ when filled with the following solution (mM): 120 KMeSO4, 15 KCl, 10 HEPES, 0.2 EGTA, 2 MgCl2, 4 Na2ATP, 0.3 Tris-GTP, 14 Tris – phosphocreatinine, 0.2% neurobiotin, pH adjusted to 7.3 with 1 M KOH (295 mOsm/l). Recordings were made using an Axoclamp 200B amplifier (Molecular Devices Ltd) and acquired using pClamp 10.4 (Molecular Devices Ltd). Data were filtered at 10 kHz and sampled at 50 kHz. 5 s long, hyperpolarizing and depolarizing square pulses were injected every 10 s either at the resting membrane potential or at a fixed potential of −-70 mV to determine the intrinsic membrane characteristics and action potentials elicited in response to depolarizing stimuli. To record action potential characteristics, short 10 ms square pulses were applied. αEPSPs were generated using the equation:

#### A = (t/τ)^∗^exp(1-t/τ)

where A and τ represent the amplitude and rise time constant respectively. τ was set to be 1 ms. In all whole-cell current-clamp experiments, series resistance was in the order of 10–20 MΩ and recordings were discarded if this changed by > 20%.

##### Voltage-clamp recordings

Whole-cell voltage-clamp recordings were obtained stellate neuron somata. The ACSF was supplemented with 1 μM tetrodotoxin, 2 mM CsCl_2_, 10 mM tetraethylammonium chloride, 0.1 mM 4-aminopyradine, 20 μM nifedipine, 0.2 μM ω-agatoxin IVA, 0.2 μM ω-conotoxin GVIA, 0.2 μΜ SNX-482 and maintained at 32–36°C. Patch pipettes were filled with an intracellular solution consisting of (mM): 120 CsCl_2_, 1 CaCl_2_, 5 MgCl_2_, 10 EGTA, 10 HEPES, 4 Na_2_ATP, 0.3 Tris-GTP, 14 Tris – phosphocreatinine, 0.2% neurobiotin and pH adjusted to 7.3 with 1 mM CsOH (295–300 mOsm/l). Recordings were obtained using an Axopatch 200 B (Molecular Devices Ltd) and acquired using pClamp 10.4 (Molecular Devices Ltd) and were corrected for liquid junction potentials. Data were filtered at 10 kHz and sampled at 50 kHz. Cells were voltage-clamped at −70 mV. To activate the T-type Ca^2+^ current, a 1s pre-pulse to −100 mV followed by 1 s pulses ranging from −90 mV to −45 mV were applied every 10 s (see Figure S5A). The inactivation protocol consisted of applying 1 s pulses from −100 mV to −55 mV followed by a 1 s step to −50 mV every 10 s^27^ (Figure S5B). A leak subtraction step consisting of a 1s step to −100 mV followed by a 1s step to −110 mV was applied between each protocol. Each protocol was repeated at least three times in the absence and after 20 min application of 100 nM TTA-P2 (Figure S5). Series resistance was in the order of 10 MΩ–30 MΩ and compensated for by a minimum of 70%. Recordings were discarded if series resistance altered by more than 10%.

#### Neurobiotin staining

Following electrophysiological recordings, all slices were fixed in 0.4% paraformaldehyde solution for a minimum of 20 min followed by a three separate washes with phosphate buffered solution (PBS). The slices were then stained with Alexa Fluor 488 streptavidin conjugate antibodies (0.2% in PBS) using a standard protocol.^50^ The slices were then mounted on microscope slides and stored at least overnight at 4°C. The slices were subsequently viewed under low magnification using a confocal microscope (Zeiss LSM 710) to confirm if the neuron recorded from was located in the dorsal or ventral mEC as in Figure S1.

#### Quantitative PCR

400 μm mEC parasagittal slices were prepared from 6 to 8 week old wildtype mice as described above. The dorsal and ventral mEC layer II/III was micro-dissected from the slices and immediately frozen on dry ice. Tissue samples were disrupted using a rotor-stator homogenizer (Disperser T10, IKA, Staufen, Germany). Total RNA was extracted from the tissue using the RNeasy Lipid Tissue Kit (Qiagen) including an on-column DNase step. Reverse transcription (RT) was performed on matched amounts of RNA from Dorsal and Ventral samples from the same animal, using High capacity RNA-to-cDNA kit (Applied Biosystems). RT-negative controls were included. TaqMan qRT-PCR (40 cycles) performed with an Applied Biosystems 7500/7500 Fast Real-Time PCR system was used to determine the relative abundance of the Ca_V_3.2 α1 subunit in pair-matched samples (between 17 and 50 ng cDNA). The following TaqMan assays with TaqMan Gene Expression Master Mix were used in accordance to the manufacturer’s protocol (gene name: assay ID): Hypoxanthine guanine phosphoribosyltransferase (*Hprt1)*: Mm00446968_m1, cacna1h (Ca_V_3.2): Mm00445382_m1. Measurements were performed in triplicate on independent RNA preparations from nine mice, and Ca_V_3.2 mRNA levels were normalised for expression of *Hprt1* mRNA. The experimenter was blind to the mouse genotype until data analysis had been completed. Sample size was determined by previous experience of similar experiments. Comparison between dorsal and ventral tissue from each mouse was made by calculating ΔΔC_T_.

#### Computational modeling

All simulations were carried out using the NEURON simulation environment (v7.7.2).^51^ For all simulations we used a reconstructed morphology of a stellate cell (courtesy of Prof. M. F. Nolan (University of Edinburgh, UK)^8^), with uniform passive properties (Cm = 1 μF/cm^2^, *R*m = 50 kΩ/cm^2^, *R*a = 150 Ω cm). Temperature was set at 34°C. Active properties included a transient Na^+^ conductance, two types of K^+^ currents (a delayed rectifier and a Ca^2+^-dependent K^+^ conductance), a non-specific I_h_ current, a Ca^2+^ conductance modeling T-type Ca^2+^ currents, and a simple Ca^2+^-extrusion mechanism with a 500 ms time constant. Kinetics for the Ca^2+^-dependent K^+^ current was taken from a previously published model (ModelDB accession no. 112546).^52^ Parameters for the other currents are reported in Table S4. All model and simulation files will be uploaded to the ModelDB database (https://senselab.med.yale.edu/modeldb/266797). Because the scope of this model was to test a specific hypothesis on the role of the interaction between T-type Ca^2+^ and persistent Na^+^ currents, rather than implement a full model for these cells, a more extensive parameter search was not performed. The peak conductance for all channels was manually adjusted following a trial-and-error procedure to reproduce the main effects observed in the experimental traces at 100 and 150pA. The effects of TTA-P2 application were modeled with a complete block of the T-type Ca^2+^ conductance.

### Quantification and statistical analysis

#### Sholl analysis, cell soma size measurements and estimation of cell location

Low magnification and high magnification acquired confocal images were uploaded into ImageJ. The total mEC length was measured from the parasubicular border (i.e. the dorsal border) to the ventral border. 30% of this was calculated and cells located within the distance from either the dorsal or ventral borders were classified as dorsal or ventral respectively.

To estimate the cell soma area using ImageJ macros, high magnification (×60) were uploaded into ImageJ. For cells that had full-length dendrites extending to the edge of the slice, Sholl analysis was carried out using high magnification images as previously described.^39^^,^^50^ Concentric circles that were 10 μm apart were constructed around the soma. The number of dendrites crossing each circle were counted for each cell (Figure S2B).

#### Whole-cell current clamp recording analysis

Clampfit 10.4 (Molecular Devices Ltd) was used. The initial peak voltage (Figure 3A) was the voltage change between the baseline and peak amplitude value for a given current pulse. Sag amplitude was measured as the difference between the peak amplitude and the steady state potential for a given current pulse (Figure 2A). R_N_ was calculated by dividing the difference in steady state voltage in the last 25 ms of a 5 s, step at −70 mV or RMP with the current injection applied. The dorsal-ventral location dependent effects of R_N_ were fitted using the following equation with OriginPro 2021 software (OriginLab):C

y = a + b^∗^x

where a is the y intercept and b is the slope of the fit. The Pearson’s correlation coefficient (r) was obtained from the fit using OriginPro software.

Action potentials elicited by 5 s depolarizing steps were counted. The latency to spike was computed as the time for the first action potential to be initiated at the smallest depolarizing step applied. The amplitude of the afterhyperpolarization following the first single action potential to be generated by the smallest positive step applied was measured as the difference between the action potential threshold and the maximal voltage deflection generated during the afterhyperpolarization.

To measure the action potential threshold of an action potential generated by a small 10 ms stept, a phase plane plot was constructed (Figure S4).^39^ For this, the voltage was differentiated with respect to time (dV/dt) and plotted against voltage. The threshold was defined as the voltage at the point of deflection for dV/dt to be greater than zero. The spike amplitude was the voltage difference between the threshold and the peak. The action potential half-width was then estimated at half the spike amplitude.

The αEPSP decay time constant (τ) was estimated by fitting the decay phase with a single exponential.

I(t) = A^∗^exp(-t/τ).

where I is the current amplitude, A is the peak amplitude, τ is the decay time constant.^53^ The summation ratio for a train of 5 αEPSPs at either 20 Hz or 50 Hz was the ratio of the amplitude of the fifth αEPSP and the first αEPSP. To estimate the summation ratio following αEPSP-spike coupling, the action potential threshold of the first spike elicited with an αEPSP was expressed as a ratio of the amplitude of the first αEPSP in the train.

#### T-type Ca^2+^ current measurements

Currents generated by the activation and inactivation protocols were first leak subtracted in Clampfit 10.4. The resulting currents in the absence of TTA-P2 were then subtracted from those obtained in the presence of TTA-P2 to obtain isolated T-type Ca^2+^ currents (Figure S5). The peak amplitudes of these were measured at all voltages. The distance from the dorsal border of a subset of neurons was estimated and plotted against the current amplitude. Linear regression analysis was carried out using OriginPro 2021 software by fitting the data to the following equation.

#### y = a + b^∗^x

where a is the y intercept and b is the slope of the fit. The Pearson’s correlation coefficient (r) was obtained from the fit using OriginPro software.

To generate the activation and inactivation curve, the peak amplitudes at different voltages were expressed as a ratio of the maximal current amplitude produced by the protocol at any given voltage (I/I_Max_). This ratio was then plotted against the voltage (Figure S6C). The curves were then fitted using OriginPro10 (OriginLab) with a Boltzmann equation:

y = A2 +(A1-A2)/(1+exp(x-x0)/k))

where A1 and A2 are the initial and final yvalues, ×0 represents the half-maximal voltage (V_1/2_) and k is the slope of the curve.

In addition, the rise and decay time constants were estimated by fitting the currents obtained with activation protocol with a single exponential function as described. These were plotted against voltages and fitted in OriginPro 2021 using a second order polynomial function of the order:

y = A + Bx + Cx^2^

where A is an offset value and B and C are coefficients.

#### Statistical analysis

Group data are expressed as mean ± SEM. Sample size was based on our previous experience for doing similar types of electrophysiology experiments. To determine statistical significance at p < 0.05 between the four groups, a two-way ANOVA with Fisher’s Least Significance Difference (LSD) post hoc test were performed using GraphPad Prism 8.0. In some cases, a two-way ANOVA adjusted for multiple comparisons using a Bonferroni Constant was performed to avoid false positives, as specified in the figure legends. To determine if there were significant differences between control conditions and following application of a T-type Ca^2+^ channel inhibitor, paired t-tests were used. Significance (p values) are stated on figures or Table S7.

## Data Availability

• All data reported in this paper will be shared by the lead contact upon request. • All original code has been deposited at ModelDB and is publicly available as of the date of publication. DOIs are listed in the key resources table. • Any additional information required to re-analyse the data reported in this paper is available from the lead contact upon request.

## Supplemental information

Document S1. Figures S1–S7 and Tables S1–S7

Document S2. Article plus supplemental information
