# Supplementary material for: T-type Ca2+ and persistent Na+ currents synergistically elevate ventral, not dorsal, entorhinal cortical stellate cell excitability
Source: Cell Rep. 2023 Jun 26;42(7):112699. doi: 10.1016/j.celrep.2023.112699 (PMC10687207; doi:10.1016/j.celrep.2023.112699)
Supplement: Document S1. Figures S1–S7 and Tables S1–S7 [file mmc1.pdf]

Cell Reports, Volume *42*

Supplemental information

T-type Ca^{2+} and persistent Na^{+} currents synergistically elevate ventral, not dorsal, entorhinal cortical stellate cell excitability

Aleksandra Topczewska, Elisabetta Giacalone, Wendy S. Pratt, Michele Migliore, Annette C. Dolphin, and Mala M. Shah

Topczewska et al., Supp Fig 1

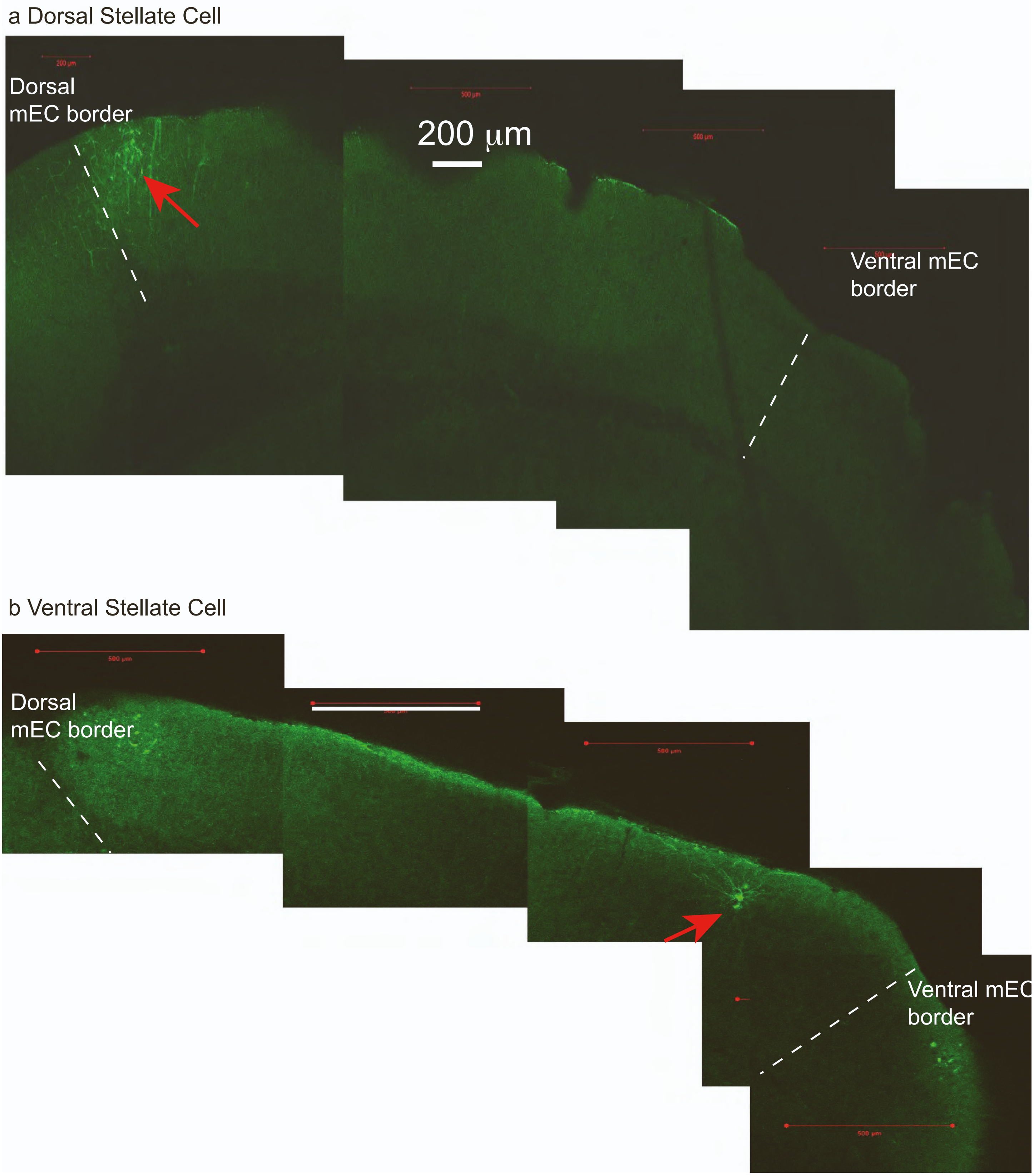

<u>Supp Fig 1 (related to Fig 1)</u>: Methodology for estimating stellate cell position within dorsal and ventral mEC borders. a, b Confocal images of a brain slice showing the location of example stellate neurons (red arrow) within the dorsal and ventral mEC borders. The dorsal border was defined as the presubiculum/ mEC border and can be clearly visualised in the slice as a dark band. The ventral mEC border was estimated as that which corresponded to the point of the CA1/subiculum border. The total distance between the dorsal and ventral borders was approximately 2500 μm, corresponding to that previously reported. Stellate cells between these borders were patched onto and filled with the dye neurobiotin. Post-hoc staining revealed the stellate cell morphology (indicated by arrow). Confocal images at low magnification (x10) were taken to reconstruct the slice. Measurements were then made to estimate the position of the recorded neuron. In **a**, the dorsal neuron is estimated to be approximately 14 % of the dorsal-ventral axis whereas in **b**, the ventral cell is approximately 80 % along the dorsal-ventral axis.

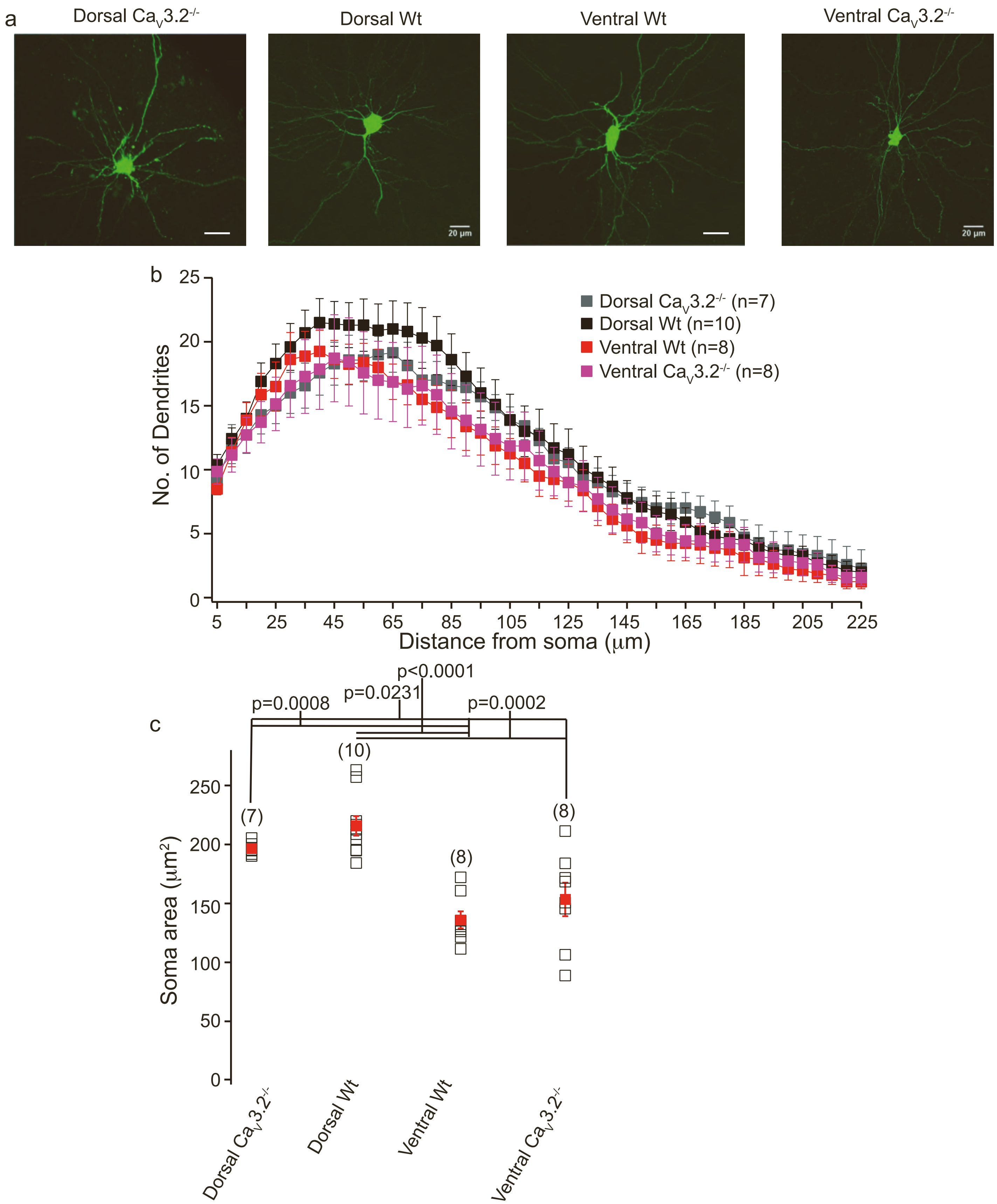

<u>Supp Fig 2 (related to Fig 1)</u>: Morphological characteristics of wildtype and Ca$_V$3.2$^{-/-}$ dorsal and ventral mEC layer II stellate neurons. **a** Confocal images of wildtype (Wt) and Ca$_V$3.2$^{-/-}$ dorsal and ventral mEC layer II stellate neurons. These were filled with neurobiotin and stained using Alexa Fluor 488 (see **Star Methods**). The scale bar shown on each image corresponds to 20 μm. **b** Sholl analyis showing the number of dendrites imaged at a given distance from the soma in wildtype and Ca$_V$3.2$^{-/-}$ dorsal and ventral neurons. Each symbol represents the mean and SEM. **C**. Soma size of dorsal and ventral mEC layer II stellate neurons. Open (black) and filled (red) squares depict individual and mean data points respectively. The numbers of observations are in parenthesis. Asterisks indicate significance at $p < 0.05$ (two way ANOVA adjusted for multiple comparisons using a Bonferroni constant).

Topczewska et al., Supp Fig 3

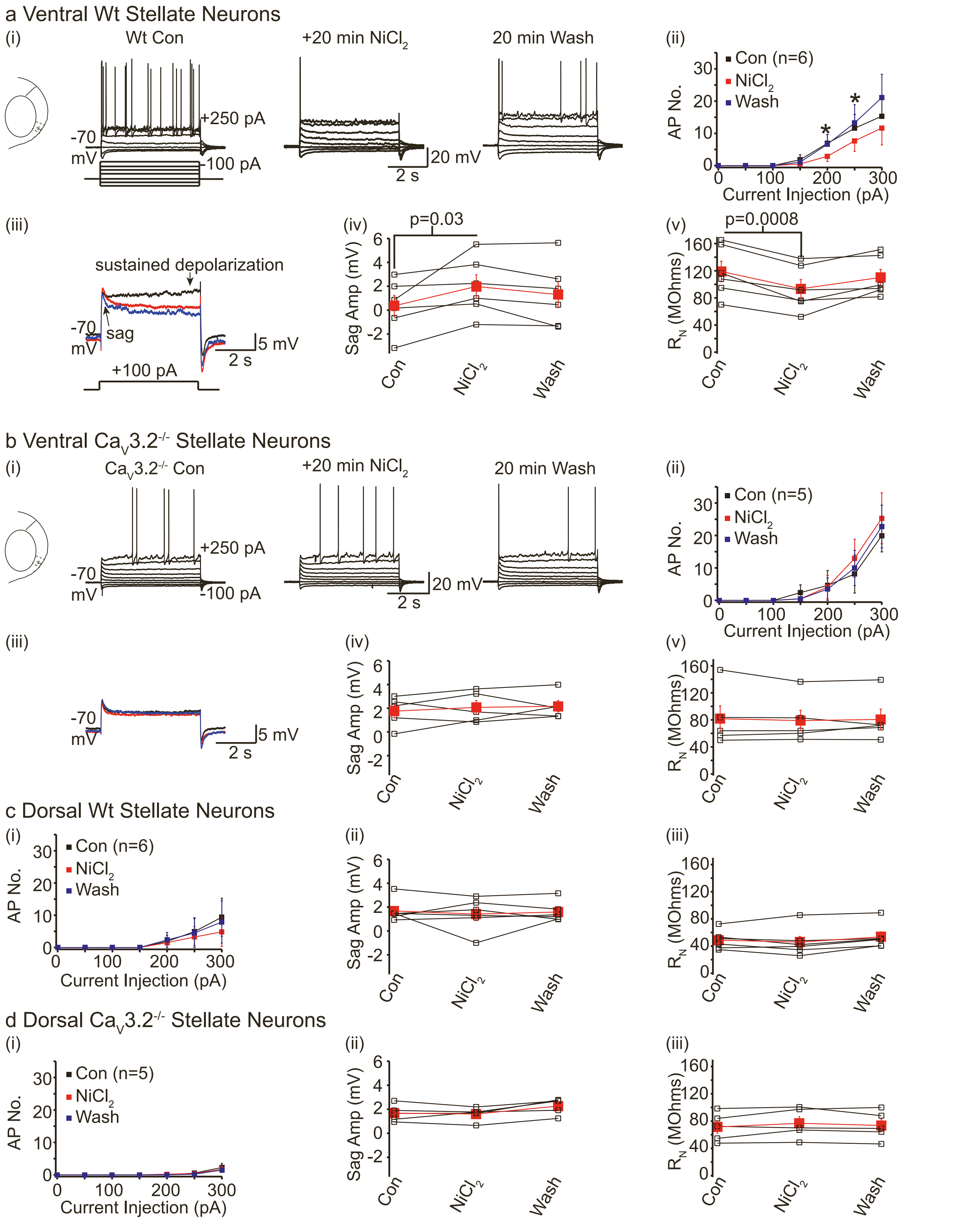

Supp Fig 3 (Related to Fig 1, Fig 2 and Fig 3): Ventral wildtype stellate neuron activity, depolarizing sag and input resistance at positive potentials is selectively affected by $NiCl_2$. a(i), b(i) Representative whole-cell current clamp recordings from ventral wildtype and $Ca_V3.2^{-/-}$ stellate neurons in the absence (con), after 20 min of $NiCl_2$ application and following 20 min washout respectively. The recordings were produced by applying 5 s long steps from -100 pA to +250 pA as shown in the schematic below the trace in **a(i)**. The scale shown in **a(i)** and **b(i)** applies to all traces within these panels. **a(ii), b(ii)** Graphs depicting the number of action potentials elicited by depolarizing steps in ventral wildtype and $Ca_V3.2^{-/-}$ stellate neurons respectively. The numbers of observations are in parenthesis. Asterisks signify significance at $p< 0.05$ when action potential numbers are compared under control conditions and in the presence of $NiCl_2$ using paired t-tests (exact p values are stated in Supp Table 7). **a(iii), b(iii)** The +100 pA traces from the recordings shown in **a(i) and b(i)** respectively on an expanded time scale. The traces have been superimposed. **a(iv), b(iv), a(v), b(v)** Plots illustrating the sag amplitude and input resistance measured using the +100 pA current pulse without $NiCl_2$, with $NiCl_2$ and following washout of $NiCl_2$ in ventral wildtype and $Ca_V3.2^{-/-}$ stellate neurons. Open black and filled red squares represent individual and mean values respectively. Control values are compared with those obtained after 20 min treatment with $NiCl_2$ using paired t-tests. The p values are stated for when a significance of $p < 0.05$ was detected. **c, d** Graphs depicting action potential numbers with incremental 5 s, depolarizing steps **(i)**, sag amplitude with the +100 pA step **(ii)** and the input resistance obtained using the +100 pA step **(iii)** in dorsal wildtype and $Ca_V3.2^{-/-}$ stellate neurons respectively in the absence, presence and following washout of $NiCl_2$. Filled squares with error bars represent mean and SEM values for all graphs while open squares depict individual values (**(ii), (iii)**).

Topczewska et al., Supp Fig 4

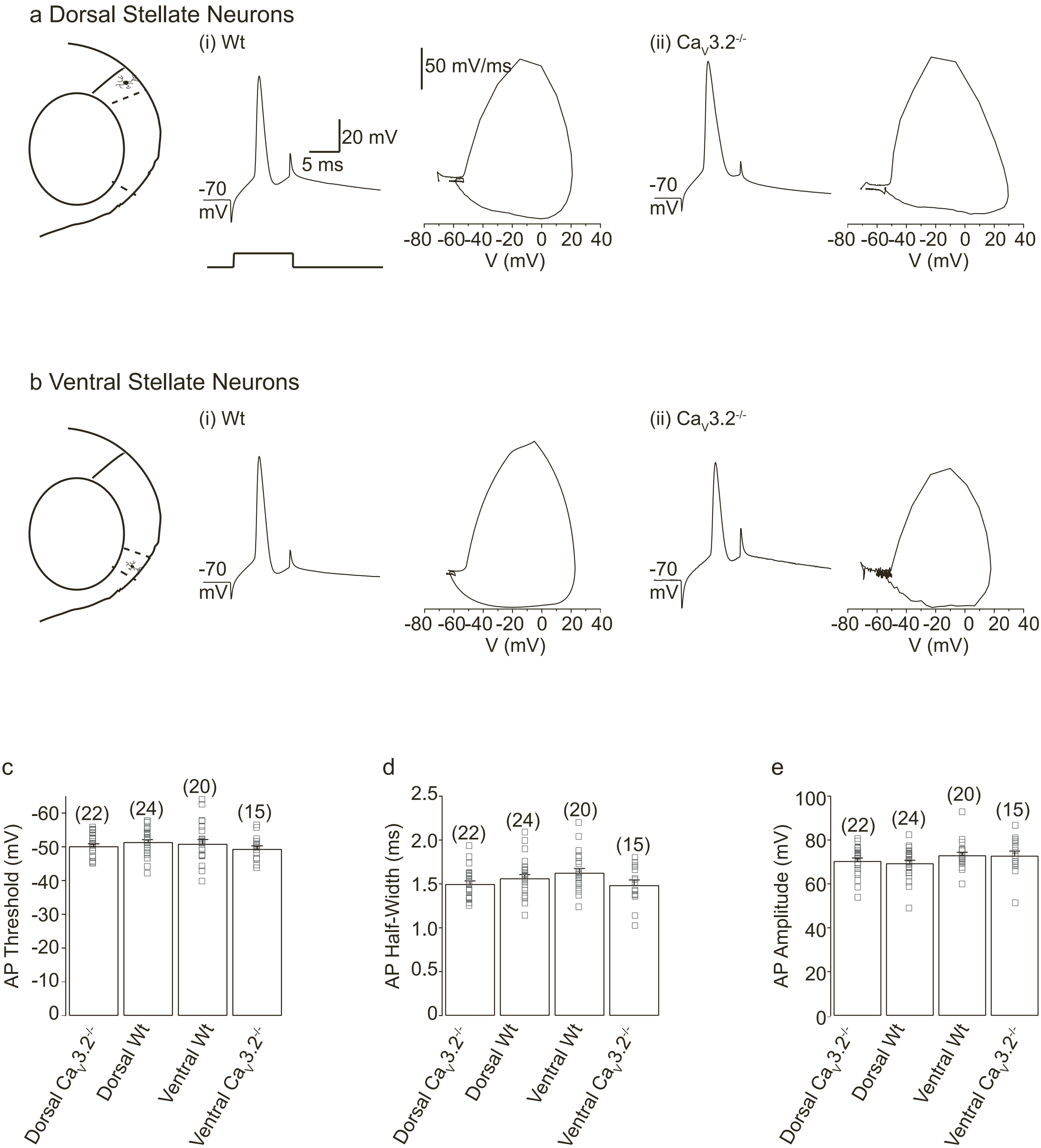

<u>Supp Fig 4 (related to Fig 1)</u>: $Ca_V3.2$ Ca^{2+} channels have little effect on action potential characteristics. **a, b** Example action potential traces from dorsal and ventral wildtype (**a(i), b(i)**) and $Ca_V3.2$ null (**a(ii), b(ii)**) stellate neurons. Individual spikes were elicited using a 10 ms step from a fixed potential of -70 mV. The phase plane plots for the individual action potentials are shown next to them. The action potential threshold was measured from these. The scale bars associated with the single action potential and phase plane plot in **a(i)** applies to all traces and plots in **a** and **b**. **c, d, e** Bar graphs depicting the mean and SEMs for action potential thrshold, half-width and action potential for all 4 groups of neurons respectively. Open squares represent individual values of the measurements for each group. The number of observations per group are shown in parenthesis above each bar. No significance at $p < 0.05$ was detected (statistics performed using a two-way ANOVA adjusted for multiple comparisons using a Bonferroni Constant).

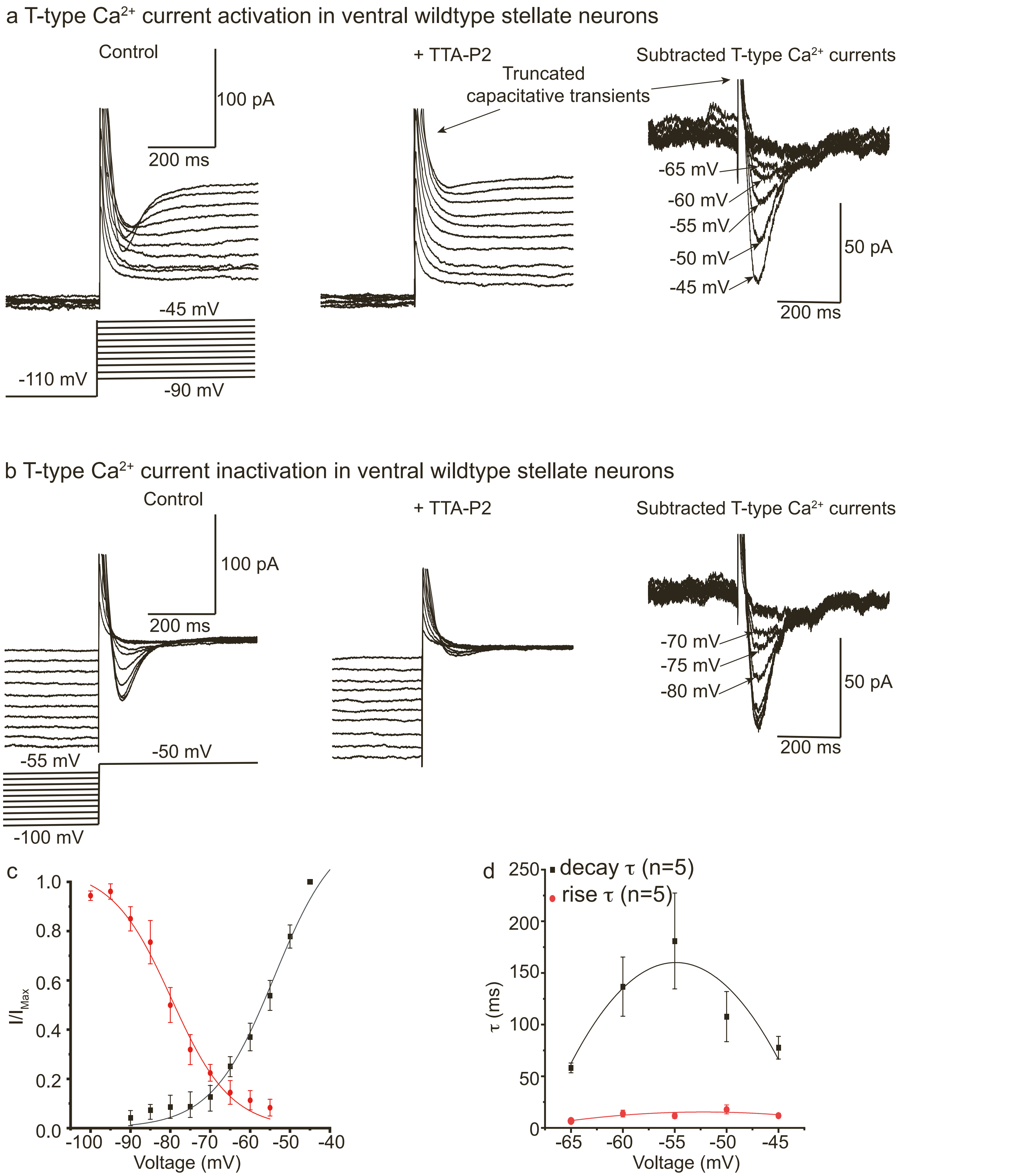

Supp Fig 5 (related to Fig 4): Biophysical properties of T-type Ca^{2+} currents in ventral wildtype stellate neurons. a. b Example raw traces of currents generated by the voltage protocols shown under control conditions and in the presence of 100 nM TTA-P2. The scale bar associated with the control trace also applies to the trace recorded with TTA-P2. All traces have been leak-subtracted (see **Star Methods**). Recordings following 20 min TTA-P2 application were subtracted from those obtained under control conditions to isolate the T-type Ca^{2+} current (far right panels). Current amplitudes, decay time constants (τ) and rise τ values were obtained from these traces. **c** The activation (black) and inactivation (red) curve of T-type Ca^{2+} currents recorded from 7 ventral wildtype stellate neurons. Each point represents the mean and SEM. The values were fitted with a Boltzmann function (see Experimental procedures). **d** A plot showing the variance of the decay and rise τ with voltage. Each point represents the mean and SEM recorded from 5 different neurons.

Topczewska et al., Supp Fig 6

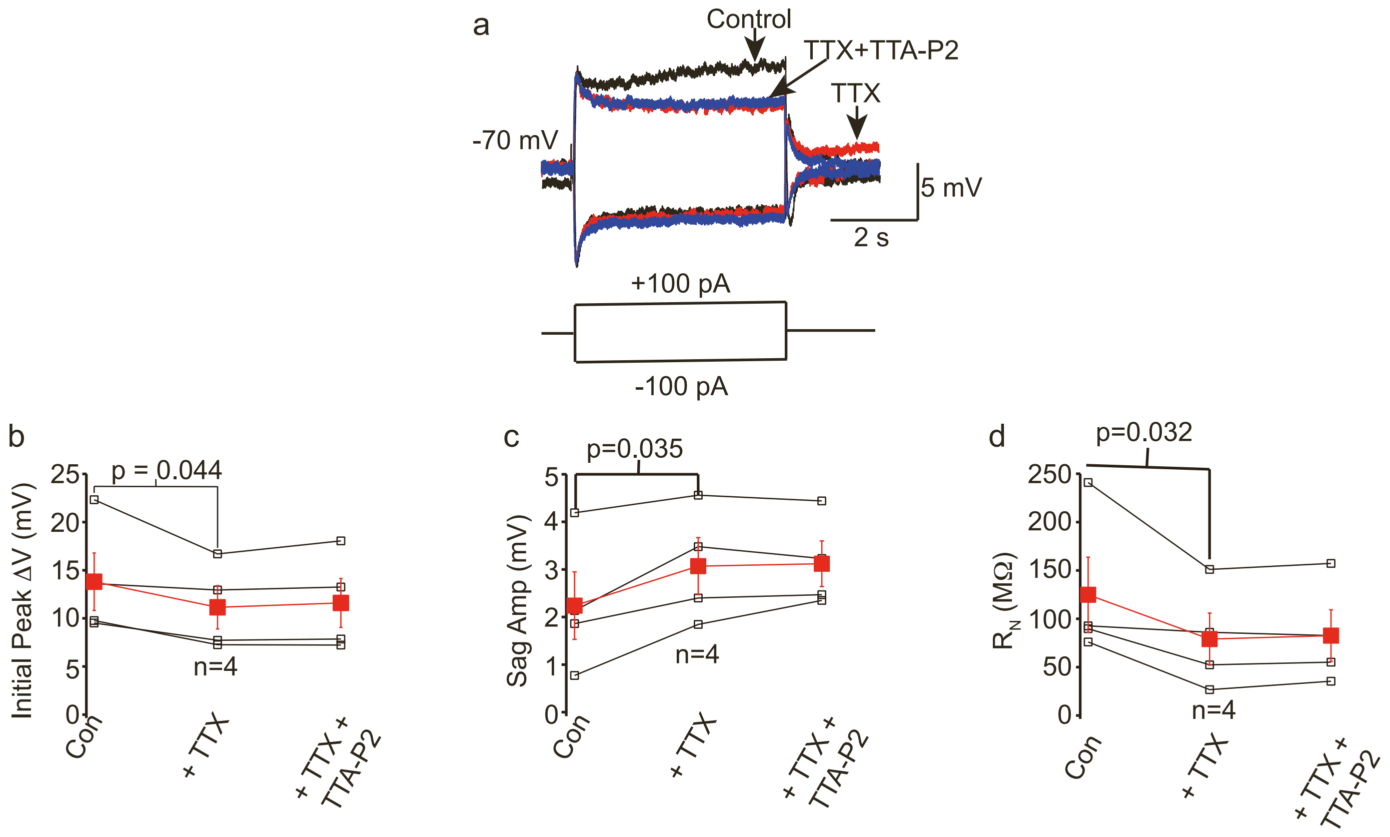

<u>**Supp Fig 6 (related to Fig 6)**</u>**: Tetrodotoxin has similar effects as riluzole on R_N a)** Example traces under control conditions (black), in the presence of the Na^+ channel inhibitor, tetrodotoxin (TTX, 1 μM, blue) and subsequent co-application of TTX and TTA-P2 (100 nM) when a -100 pA hyperpolarizing and +100 pA depolarizing steps were applied at a fixed potential of -70 mV. The traces have been superimposed. **b), c) and d)** Individual (open squares) and average (closed, red squares) initial depolarizing peak change (Δ), sag amplitude (Amp) and input resistance (R_N) values obtained when +100 pA depolarizing step at a potential of -70 mV was applied. The numbers of observations are shown on each graph.

Topczewska et al., Supp Fig 7

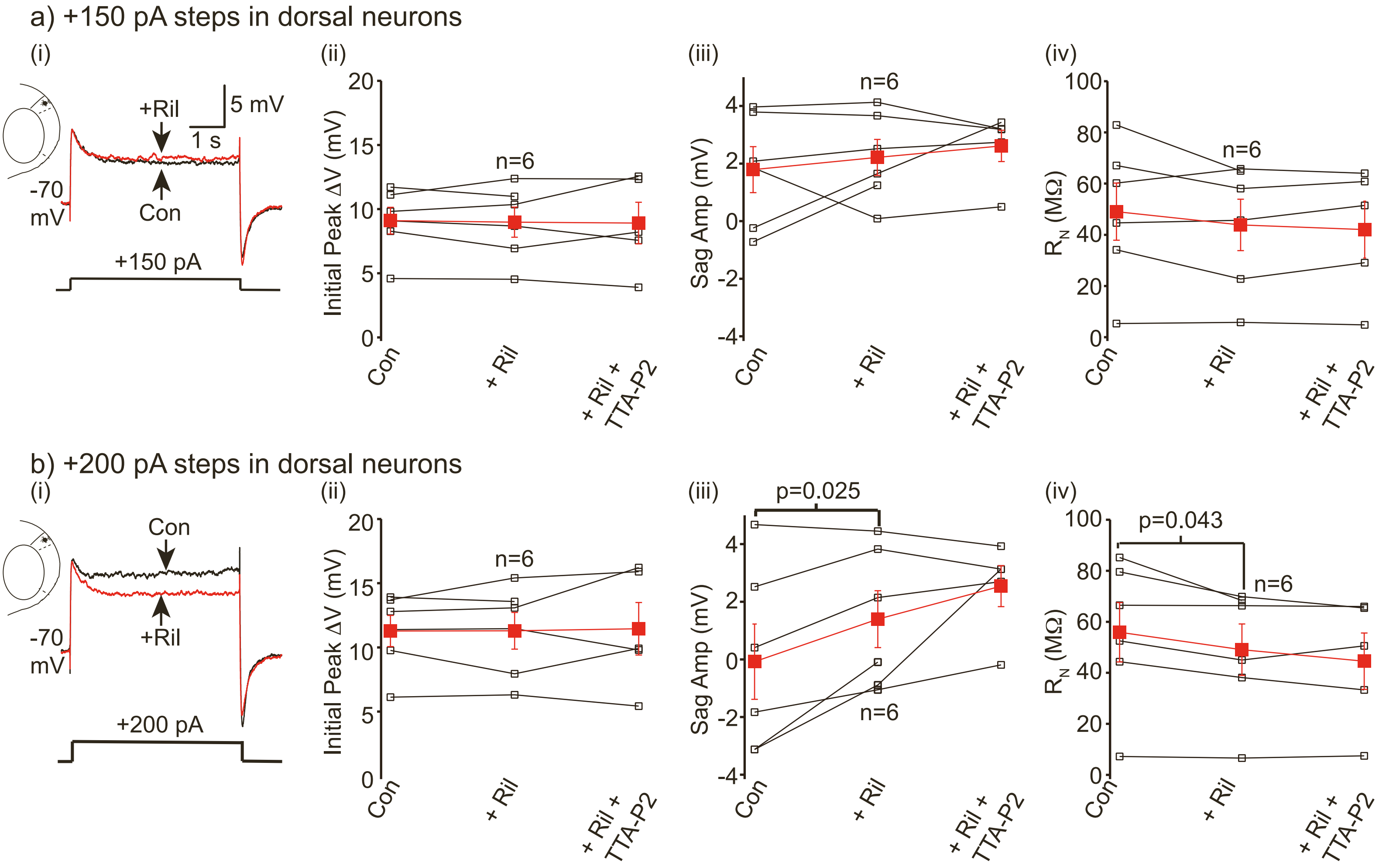

Supp Fig 7 (related to Fig 6): Persitent Na^{+} currents are activated by large depolarizing currents in dorsal stellate neurons. a(i), b(i) Example traces from dorsal stellate neurons when +150 pA and +200 pA steps were applied under control conditions (Con, black) and in the presence of riluzole (Ril, 10 μM, red). The scale shown in a(i) applies to traces in b(i) too. **a(ii), a(iii), a(iv)**, **b(ii), b(iii), b(iv)** The initial peak voltage change (Δ), sag amplitude (amp) and input resistance (R_N) values when 150 pA or 200 pA steps were applied in the absence of riluzole (Con), in the presence of riluzole and after subsequent co-application of riluzole and TTA-P2. Individual points are shown as black squares whereas mean points are shown as filled red squares. The numbers of observations are shown in parenthesis.

Supplemental Table 1

	Dorsal $Ca_V3.2^{-/-}$	Dorsal Wt	Ventral Wt	Ventral $Ca_V3.2^{-/-}$
RMP	-68.96 ± 0.43 mV (n=27)	-67.95 ± 0.54 mV (n=39)	-69.80 ± 0.36 mV (n=39)	-69.16 ± 0.56 mV (n=27)
Time to spike at RMP	1684.5 ± 263.0 ms (n=27)	1604.4 ± 173.5 ms (n=39)	1215.1 ± 179.5 ms (n=39)	1408.9 ± 221.8 ms (n=27)
Time to spike at -70 mV	2029.2 ± 292.5 ms (n=27)	1893.9 ± 205.9 ms (n=39)	1534.6 ± 241.3 ms (n=39)	1396.4 ± 232.3 ms (n=27)
R_N with -100 pA step at -70 mV	41.35 ± 2.89 MΩ (n=27)	37.62 ± 2.82 MΩ (n=39)	54.54 ± 3.44 MΩ (n=39)*	45.10 ± 3.95 MΩ (n=27)
Sag with -100 pA step at -70 mV	3.24 ± 0.30 mV (n=27)	3.09 ± 0.19 mV (n=39)	4.67 ± 0.33 mV (n=39)	3.98 ± 0.36 mV (n=27)
R_N with -100 pA step at RMP	43.27 ± 3.17 MΩ (n=27)	43.00 ± 2.89 MΩ (n=39)	56.56 ± 3.89 MΩ (n=39)*	46.43 ± 4.60 MΩ (n=27)
Sag with -100 pA step at RMP	3.34 ± 0.34 mV (n=27)	3.19 ± 0.22 mV (n=39)	4.64 ± 0.34 mV (n=39)	4.03 ± 0.39 mV (n=27)
R_N with +50 pA step at RMP	60.54 ± 4.23 MΩ (n=27)	62.83 ± 3.60 MΩ (n=39)	91.68 ± 5.55 MΩ (n=39)	67.07 ± 4.23 MΩ (n=27)
Sag with +50 pA step at RMP	1.17 ± 0.15 mV (n=27)	1.16 ± 0.11 mV (n=39)	1.28 ± 0.23 mV (n=39)	1.12 ± 0.41 mV (n=27)
Sag with +50 pA step at -70 mV	1.29 ± 0.12 mV (n=27)	1.36 ± 0.09 mV (n=39)	1.36 ± 0.19 mV (n=39)	1.48 ± 0.21 mV (n=27)
Initial Peak Voltage with +50 pA step at -70 mV	4.01 ± 0.19 mV (n=27)	3.82 ± 0.18 mV (n=39)	5.57 ± 0.29 mV (n=39)*	4.68 ± 0.24 mV (n=27)
R_N with +100 pA step at RMP	69.74 ± 4.91 MΩ (n=27)	74.81 ± 4.48 MΩ (n=39)	115.02 ± 6.77 MΩ (n=39)*	85.15 ± 5.44 MΩ (n=27)
Sag with +100 pA step at RMP	1.07 ± 0.32 mV (n=27)	1.29 ± 0.15 mV (n=39)	-0.32 ± 0.39 mV (n=39)*	1.41 ± 0.38 mV (n=27)
Sag with +100 pA step at -70 mV	1.54 ± 0.26 mV (n=27)	1.87 ± 0.17 mV (n=39)	-0.17 ± 0.38 mV (n=39)*	1.91 ± 0.32 mV (n=27)
Initial Peak Voltage with +100 pA step at -70 mV	7.93 ± 0.36 mV (n=27)	7.70 ± 0.38 mV (n=39)	10.78 ± 0.57 mV (n=39)*	9.44 ± 0.49 mV (n=27)
Afterhyperpolarization following 1st action potential elicited using a 5 s step at -70 mV	13.82 ± 0.44 mV (n=27)	14.01± 0.36 mV (n=39)	14.99 ± 0.44 mV (n=39)	15.93 ± 0.41 mV (n=27)

Supp Table 1 (Related to Fig 1): Effects of $Ca_V3.2$ currents on the RMP, initial peak voltages, sag, R_N, afterhyperpolarization following an action potential and the time to

initiate an action potential. The table depicts the resting membrane potential values (RMP) as well as the membrane potential sag and input resistance (R_N) values obtained at RMP or a fixed potential of -70 mV when a 5 s hyperpolarizing or depolarizing pulses of various amplitudes were applied. In addition, the values for the time taken for the first action potential to be generated produced with the smallest 5 s long depolarizing current step at RMP and at a fixed potential of -70 mV in all 4 groups of neurons are presented. The the amplitude of the afterhyperpolarization following the first action potential produced with the smallest depolarizing step from a fixed potential of -70 mV are also reported. * indicates significance at $p < 0.05$ when compared with dorsal wildtype or dorsal/ventral $Ca_V3.2^{-/-}$ values (statistics were performed using a two-way ANOVA adjusted for multiple comparisons using a Bonferroni Constant). A $p < 0.0001$ was obtained for comparison between ventral wildtype and ventral $Ca_V3.2^{-/-}$, dorsal wildtype or dorsal $Ca_V3.2^{-/-}$ sag amplitudes at +100 pA at -70 mV. When the sag was measured using +100 pA step at RMP, p values of 0.0014, 0.0010 and 0.011 were obtained when ventral wildtype sag amplitudes were compared with ventral $Ca_V3.2^{-/-}$, dorsal wildtype or dorsal $Ca_V3.2^{-/-}$ sag amplitudes. Values of p=0.3394, p=0.0309 and p=0.0773 were obtained when R_N measured using -100 pA step at -70 mV in ventral wildtypes were compared with that from ventral $Ca_V3.2^{-/-}$, dorsal wildtype and dorsal $Ca_V3.2^{-/-}$. Values of p=0.2961, p=0.0008 and p=0.03866 were obtained when R_N obtained with -100 pA step at RMP in ventral wildtype were compared with that from ventral $Ca_V3.2^{-/-}$, dorsal wildtype and dorsal $Ca_V3.2^{-/-}$. Values of p=0.0020 and p=0.0019 were obtained when R_N measured using +100 pA and +50 pA steps respectively were compared between ventral wildtype and $Ca_V3.2^{-/-}$ neurons. p<0.0001 was obtained when R_N measurements obtained with either +100 pA or +50 pA steps at RMP were compared between ventral wildtype and dorsal wildtype or $Ca_V3.2^{-/-}$ neurons. Whilst initial peak voltages obtained with +50 pA or +100 pA steps with ventral wildtype and $Ca_V3.2^{-/-}$ neurons were similar, $p < 0.001$ values were obtained when initial peak voltages were compared between ventral wildtype and dorsal wildtype or $Ca_V3.2^{-/-}$ neurons.

Supplemental Table 2

	Dorsal $Ca_V3.2^{-/-}$	Dorsal Wt	Ventral Wt	Ventral $Ca_V3.2^{-/-}$
RMP in the absence of TTA-P2	-70.29 ± 0.34 mV (n=5)	-71.68 ± 0.32 mV (n=6)	-70.22 ± 0.37 mV (n=5)	-69.96 ± 1.13 mV (n=6)
RMP in the presence of TTA-P2	-71.13 ± 0.30 mV (n=5, p=0.11)	-71.50 ± 0.54 mV (n=6, p=0.46)	-71.91 ± 0.79 mV (n=5, p=0.09)	-70.39 ± 1.17 mV (n=6, p=0.25)
RMP in the absence of $NiCl_2$	-70.72 ± 0.38 mV (n=5)	-71.49 ± 0.57 mV (n=5)	-71.11 ± 0.47 mV (n=6)	-70.69 ± 0.42 mV (n=3)
RMP in the presence of $NiCl_2$	-71.17 ± 0.52 mV (n=5, p=0.57)	-71.32 ± 0.80 mV (n=5)	-71.65 ± 0.32 mV (n=6)	-71.44 ± 0.25 mV (n=3, p=0.16)
Sag with -100 pA step in the absence of TTA-P2	3.51 ± 0.78 mV (n=6)	3.33 ± 0.4 mV (n=7, p=0.86)	3.4 ± 0.21 mV (n=7, p=0.39)	4.19 ± 0.71 mV (n=7)
Sag with -100 pA step in the presence of TTA-P2	3.24 ± 0.68 mV (n=6, p=0.21)	3.22 ± 0.23 mV (n=7, p=0.61)	3.18 ± 0.27 mV (n=7, p=0.36)	4.32 ± 0.68 mV (n=7, p=0.46)
Sag with -100 pA step in the absence of $NiCl_2$	3.05 ± 0.30 mV (n=5)	2.55 ± 0.34 mV (n=6)	4.22 ± 0.84 mV (n=6)	3.50 ± 0.57 mV (n=5)
Sag with -100 pA step in the presence of $NiCl_2$	3.25 ± 0.33 mV (n=5, p=0.20)	2.21 ± 0.28 mV (n=6, p=0.21)	3.85 ± 0.84 mV (n=6, p=0.06)	3.46 ± 0.51 mV (n=5, p=0.67)
R_N with -100 pA step in the absence of TTA-P2	43.68 ± 4.78 MΩ (n=6)	36.48 ± 4.17 MΩ (n=7)	52.17 ± 5.05 MΩ (n=7)	49.88 ± 12.16 MΩ (n=7)
R_N with -100 pA step in the presence of TTA-P2	48.29 ± 4.86 MΩ (n=6, p=0.34)	35.07 ± 4.09 MΩ (n=7, p=0.73)	50.86 ± 6.63 MΩ (n=7, p=0.72)	53.43 ± 9.08 MΩ (n=7, p=0.66)
R_N with -100 pA step in the absence of $NiCl_2$	48.51 ± 6.28 MΩ (n=5)	35.63 ± 2.78 MΩ (n=6)	65.85 ± 10.74 MΩ (n=6)	45.93 ± 4.17 MΩ (n=5)
R_N with -100 pA step in the presence of $NiCl_2$	51.86 ± 7.16 MΩ (n=5, p=0.19)	32.18 ± 4.12 MΩ (n=6, p=0.17)	66.22 ± 9.38 MΩ (n=6, p=0.92)	55.15 ± 6.75 MΩ (n=5, p=0.17)
Initial peak depolarization with +50 pA	4.32 ± 0.22 mV (n=6)	3.91 ± 0.29 mV (n=7)	4.63 ± 0.48 mV (n=7)	4.77 ± 0.45 mV (n=6)

step without TTA-P2 (con)				
Initial peak depolarization with +50 pA step with TTA-P2	4.37 ± 0.41 mV (n=6, p=0.85)	3.67 ± 0.24 mV (n=7, p=0.31)	4.47 ± 0.42 mV (n=7, p=0.32)	5.18 ± 0.42 mV (n=6, p=0.18)
Sag with +50 pA without TTA-P2 (con)	1.25 ± 0.32 mV (n=6)	1.51 ± 0.15 mV (n=7)	0.37 ± 0.3 mV (n=7)	1.14 ± 0.64 mV (n=6)
Sag with +50 pA with TTA-P2	1.20 ± 0.37 mV (n=6, p=0.83)	1.32 ± 0.13 mV (n=7, p=0.11)	1.05 ± 0.19 mV (n=7, p=0.002)	1.24 ± 0.66 mV (n=6, p=0.67)
R_N with +50 pA step in the absence of $NiCl_2$	61.33 ± 8.18 MΩ (n=5)	44.01 ± 3.29 MΩ (n=6)	103.76 ± 15.71 MΩ (n=6)	65.58 ± 9.49 MΩ (n=5)
R_N with +50 pA step in the presence of $NiCl_2$	67.23 ± 8.51 MΩ (n=5, p=0.27)	41.40 ± 7.01 MΩ (n=6, p=0.55)	77.32 ± 13.80 MΩ (n=6, p=0.0007)	69.31 ± 11.05 MΩ (n=5, p=0.38)
Initial peak depolarization with +50 pA step without $NiCl_2$ (con)	4.29 ± 0.44 mV (n=5)	3.24 ± 0.22 mV (n=6)	6.20 ± 0.49 mV (n=6)	4.69 ± 0.58 mV (n=5)
Initial peak depolarization with +50 pA step with $NiCl_2$	4.45 ± 0.46 mV (n=5, p=0.33)	2.95 ± 0.27 mV (n=6, p=0.01)	5.98 ± 0.42 mV (n=6, p=0.08)	5.02 ± 0.69 mV (n=5, p=0.28)
Sag with +50 pA without $NiCl_2$ (con)	1.33 ± 0.09 mV (n=5)	1.08 ± 0.13 mV (n=6)	1.41 ± 0.48 mV (n=6)	1.46 ± 0.21 mV (n=5)
Sag with +50 pA with $NiCl_2$	1.24 ± 0.23 mV (n=5, p=0.74)	0.95 ± 0.19 mV (n=6, p=0.28)	1.84 ± 0.48 mV (n=6, p=0.02)	1.56 ± 0.28 mV (n=5, p=0.58)
Initial peak depolarization with +100 pA step without TTA-P2 (con)	8.47 ± 0.48 mV (n=6)	7.88 ± 0.62 mV (n=7)	8.58 ± 0.98 mV (n=7)	9.41 ± 0.96 mV (n=6)
Initial peak depolarization with +100 pA step with TTA-P2	8.79 ± 0.78 mV (n=6, p=0.64)	7.38 ± 0.53 mV (n=7, p=0.36)	8.98 ± 1.03 mV (n=7, p=0.26)	10.4 ± 1.01 mV (n=6, p=0.053)
Sag with +100 pA without TTA-P2 (con)	1.29 ± 0.51 mV (n=6)	1.97 ± 0.38 mV (n=7)	-2.07 ± 0.86 mV (n=7)	1.60 ± 0.71 mV (n=6)

Sag with +100 pA with TTA-P2	1.95 ± 0.51 mV (n=6, p=0.58)	1.72 ± 0.19 mV (n=7, p=0.61)	0.78 ± 0.57 mV (n=7, p=0.0035)	1.81 ± 0.81 mV (n=6, p=0.66)
R_N with +100 pA step in the absence of $NiCl_2$	71.38 ± 9.29 MΩ (n=5)	48.66 ± 5.62 MΩ (n=6)	118.96 ± 15.11 MΩ (n=6)	81.71 ± 18.93 MΩ (n=5)
R_N with +100 pA step in the presence of $NiCl_2$	76.75 ± 9.78 MΩ (n=5, p=0.16)	45.92 ± 8.50 MΩ (n=6, p=0.49)	93.46 ± 13.53 MΩ (n=6, p=0.002)	78.98 ± 15.28 MΩ (n=5, p=0.51)
Initial peak depolarization with +100 pA step without $NiCl_2$ (con)	8.84 ± 0.82 mV (n=5)	6.44 ± 0.44 mV (n=6)	11.84 ± 0.80 mV (n=6)	9.59 ± 1.14 mV (n=5)
Initial peak depolarization with +100 pA step with $NiCl_2$	8.85 ± 0.96 mV (n=5, p=0.99)	6.10 ± 0.41 mV (n=6, p=0.12)	11.39 ± 0.99 mV (n=6, p=0.15)	10.17 ± 1.29 mV (n=5, p=0.26)
Sag with +100 pA without $NiCl_2$ (con)	1.67 ± 0.31 mV (n=6)	1.68 ± 0.38 mV (n=6)	0.36 ± 0.88 mV (n=6)	1.76 ± 0.56 mV (n=5)
Sag with +100 pA with $NiCl_2$	1.61 ± 0.26 mV (n=6, p=0.74)	1.42 ± 0.55 mV (n=6, p=0.66)	1.98 ± 0.98 mV (n=6, p=0.03)	2.08 ± 0.57 mV (n=5, p=0.23)

Supp Table 2 (Related to Fig 1, Fig 2 and Fig 3): The effect of T-type Ca^{2+} channel inhibitors on membrane properties of dorsal and ventral wildtype and $Ca_V3.2^{-/-}$ stellate neurons. The table depicts the resting membrane potential (RMP), initial peak voltage change, sag amplitude and R_N values obtained using a 5 s steps from a holding potential of -70 mV when current pulses of -100 pA, +50 pA or +100 pA were applied in the absence and presence of the T-type Ca^{2+} channel inhibitors, TTA-P2 (100 nM) and $NiCl_2$ (50 μM) in dorsal and ventral wildtype (Wt) and $Ca_V3.2_{-/-}$ stellate neurons. The numbers of observations for the values for each group are depicted in parenthesis. Paired t-tests were performed to assess significance between control values and those in the presence of the T-type Ca^{2+} channel inhibitor for each set of neurons. The p values obtained are indicated in brackets.

Supplemental Table 3

	Dorsal $Ca_V3.2^{-/-}$	Dorsal Wt	Ventral Wt	Ventral $Ca_V3.2^{-/-}$
Threshold in the absence of TTA-P2	-50.38 ± 1.57 mV (n=5)	-50.48 ± 1.22 mV (n=4)	-48.45 ± 0.69 mV (n=6)	-48.87 ± 2.23 mV (n=5)
Threshold in the presence of TTA-P2	-50.58 ± 1.63 mV (n=5)	-50.61 ± 1.01 mV (n=4)	-50.31 ± 1.25 mV (n=6)	-50.62 ± 2.10 mV (n=5)
Threshold in the absence of $NiCl_2$	-48.01 ± 2.09 mV (n=4)	-51.55 ± 2.16 mV (n=4)	-49.50 ± 4.48 mV (n=4)	-47.70 ± 1.12 mV (n=3)
Threshold in the presence of $NiCl_2$	-47.98 ± 2.36 mV (n=4)	-53.73 ± 1.41 mV (n=4)	-49.59 ± 2.50 mV (n=4)	-50.97 ± 1.62 mV (n=3)
Half-width in the absence of TTA-P2	1.48 ± 0.09 ms (n=5)	1.52 ± 0.14 ms (n=4)	1.54 ± 0.10 ms (n=6)	1.49 ± 0.15 ms (n=5)
Half-width in the presence of TTA-P2	1.62 ± 0.09 ms (n=5)	1.49 ± 0.10 ms (n=4)	1.62 ± 0.09 ms (n=6)	1.63 ± 0.22 ms (n=5)
Half-width in the absence of $NiCl_2$	1.45 ± 0.04 ms (n=4)	1.62 ± 0.13 ms (n=4)	1.79 ± 0.10 ms (n=4)	1.57 ± 0.14 ms (n=3)
Half-width in the presence of $NiCl_2$	1.56 ± 0.03 ms (n=4)	1.73 ± 0.14 ms (n=4)	1.88 ± 0.08 ms (n=4)	1.77 ± 0.20 ms (n=3)
Amplitude in the absence of TTA-P2	74.29 ± 1.80 mV (n=5)	72.34 ± 1.09 mV (n=4)	76.01 ± 3.45 mV (n=6)	75.07 ± 3.52 mV (n=5)
Amplitude in the presence of TTA-P2	74.50 ± 1.46 mV (n=5)	73.35 ± 1.56 mV (n=4)	75.57 ± 3.56 mV (n=6)	74.96 ± 3.86 mV (n=5)
Amplitude in the absence of $NiCl_2$	67.86 ± 3.33 mV (n=4)	73.05 ± 0.71 mV (n=4)	72.65 ± 2.80 mV (n=4)	72.24 ± 3.73 mV (n=3)
Amplitude in the presence of $NiCl_2$	69.39 ± 4.10 mV (n=4)	72.70 ± 0.72 mV (n=4)	71.76 ± 3.04 mV (n=4)	68.87 ± 7.44 mV (n=3)

Supp Table 3 (Related to Fig 1): T-type Ca^{2+} channel inhibitors had little effect on action potential characteristics in dorsal and ventral wildtype and $Ca_V3.2^{-/-}$ stellate neurons. Single action potentials were initiated using a single 10 ms step (see Supp Fig 2) in dorsal and ventral wildtype (Wt) and $Ca_V3.2^{-/-}$ stellate neurons before and after application of T-type Ca^{2+} channel inhibitors, TTA-P2 (100 nM) and $NiCl_2$ (50 µM). Phase plane plots were made to measure the threshold of these single spikes (Supp Fig 2). The amplitude was measured from the threshold to the peak of the action potential. The half-width was measured at the point of half the amplitude of the action potential. The table shows these values in the absence and presence of TTA-P2 and $NiCl_2$ in dorsal and ventral wildtype and $Ca_V3.2^{-/-}$ stellate neurons. The numbers of observations are shown in parenthesis. Paired t-tests were

performed to assess significance between values obtained under control conditions and in the presence of a specific T-type Ca^{2+} channel inhibitor for each group of neurons. No significance at $p < 0.05$ was found.

Supplementary Table 4.

Conductance	Peak Conductance (S/cm^2)	$V_{1/2}$ act. (mV)	k act. (mV)	$V_{1/2}$ inact. (mV)	k inact. (mV)	Location
Na^+	0.01/ 0.006/ 0.006*	-30	7.2	-50	4	Axon, soma and dendrites
Delayed rectifier type K^+	0.01/0.0013/0.0013*	13	13	-	-	Axon, soma and dendrites
Persistent Na^+	0.0007/0.00025**	-58	2	-48.8	10	Axon and soma
T-type Ca^{2+} current	0.0004	-58	7.5	-55	5.44	soma
I*h*	0.0003	-71	-8	-	-	soma
M-current	0.003/0.001**	-40	-10			Axon and Soma

Supplementary Table 4 (Related to Fig 5): Properties of the selected voltage-dependent conductances used in our computational model. The complete set of files are available on ModelDB (https://senselab.med.yale.edu/modeldb/).
*values refer to peak conductance in axon, soma, and dendrites, respectively
**values refer to peak conductance in axon and soma, respectively

Supplemental Table 5

	Dorsal Wt	Ventral Wt	Ventral $Ca_V3.2^{-/-}$
RMP in the absence of Riluzole	-67.10 ± 2.21 mV (n=6)	-69.84 ± 1.14 mV (n=6)	-68.20 ± 1.64 mV (n=6)
RMP in the presence of riluzole	-67.49 ± 1.15 mV (n=6, p=0.43)	-70.68 ± 1.17 mV (n=6, p=0.33)	-69.16 ± 1.59 mV (n=6, p=0.05)
RMP in the presence of riluzole + TTA-P2	-68.22 ± 2.33 mV (n=4, p=0.10)	-70.85 ± 0.88 mV (n=6, p=0.50)	-68.67 ± 1.79 mV (n=6, p=0.21)
Sag amplitude with -100 pA step in the absence of riluzole	3.16 ± 0.44 mV (n=6)	6.43 ± 1.28 mV (n=6)	3.98 ± 1.24 mV (n=6)
Sag amplitude with -100 pA step in the presence of riluzole	3.03 ± 0.40 mV (n=6, p=0.44)	6.31 ± 1.02 mV (n=6, p=0.20)	3.68 ± 1.10 mV (n=6, p=0.45)
Sag amplitude with -100 pA step in the presence of riluzole + TTA-P2	2.72 ± 0.33 mV (n=5, p=0.21)	5.91 ± 0.99 mV (n=6, p=0.24)	3.33 ± 1.07 mV (n=6, p=0.10)
R_N with -100 pA step in the absence of riluzole	40.29 ± 7.08 MΩ (n=6)	47.01 ± 7.04 MΩ (n=6)	45.05 ± 6.99 MΩ (n=6)
R_N with -100 pA step in the presence of riluzole	43.19 ± 8.72 MΩ (n=6, p=0.24)	47.92 ± 3.96 MΩ (n=6, p=0.84)	47.62 ± 2.75 MΩ (n=6, p=0.51)
R_N with -100 pA step in the presence of riluzole + TTA-P2	48.51 ± 10.46 MΩ (n=5, p=0.05)	51.64 ± 5.41 MΩ (n=6, p=0.22)	55.05 ± 2.66 MΩ (n=6, p=0.15))
Initial peak voltage change with +50 pA step in the absence of riluzole	3.11 ± 0.38 mV (n=6)	7.61 ± 0.81 mV (n=6)	4.87 ± 0.59 mV (n=6)
Initial peak voltage change	3.15 ± 0.35 mV (n=6, p=0.72)	6.90 ± 0.76 mV (n=6, p = 0.003)	4.82 ± 0.48 mV (n=6, p=0.85)

with +50 pA step in the presence of riluzole			
Initial peak voltage change with +50 pA step in the presence of riluzole and TTA-P2	2.95 ± 0.50 mV (n=6, p=0.82)	6.93 ± 0.79 mV (n=6, p = 0.79)	4.99 ± 0.48 mV (n=6, p=0.68)
Sag amplitude with +50 pA step in the absence of riluzole	1.47 ± 0.17 mV (n=6)	1.58 ± 0.34 mV (n=6)	1.76 ± 0.76 mV (n=6)
Sag amplitude with +50 pA step in the presence of riluzole	1.32 ± 0.14 mV (n=6, p=0.15)	2.83 ± 0.53 mV (n=6, 0.10)	1.58 ± 0.60 mV (n=6, p=0.41)
Sag amplitude with +50 pA step in the presence of riluzole and TTA-P2	1.27 ± 0.17 mV (n=5. p=0.21)	2.67 ± 0.55 mV (n=6, p=0.06)	1.63 ± 0.52 mV (n=6, p=0.62)
R_N with +50 pA step in the absence of riluzole	33.15 ± 9.09 MΩ (n=6)	100.00 ± 10.89 MΩ (n=6)	65.07 ± 8.35 MΩ (n=6)
R_N with +50 pA step in the presence of riluzole	36.65 ± 9.01 MΩ (n=6, p=0.33)	75.16 ± 5.57 MΩ (n=6, p=0.02)	64.82 ± 4.58 MΩ (n=6, p=0.96)
R_N with +50 pA step in the presence of riluzole and TTA-P2	33.46 ± 8.97 MΩ (n=6, p=0.08)	78.72 ± 8.67 MΩ (n=6, p=0.03)	67.15 ± 2.81 MΩ (n=6, p=0.77)

<u>Supp Table 5 (Related to Fig 6):</u> Ca$_V$3 currents together with subthreshold Na^+ currents have little effect on the resting membrane potential or R_N and sag generated with hyperpolarizing pulses in ventral or dorsal stellate neurons. The effects of riluzole and co-application of riluzole (10 μM) and TTA-P2 (100 nM) on the resting membrane potential (RMP) compared with under control conditions in dorsal wildtype, ventral wildtype and ventral Ca$_V$3.2$^{-/-}$ neurons is tabulated. In addition, the R_N and sag amplitude generated with a -100 pA hyperpolarizing pulse as well as the initial peak voltage change, sag amplitude and R_N produced by injecting +50 pA current pulses in the absence of any drug (control), in the presence of riluzole and subsequent co-application of riluzole and TTA-P2 are shown. These

measurements were all done at a fixed potential of -70 mV to allow comparison between neurons. Paired t-tests comparing the values in the presence of riluzole or co-application of riluzole and TTA-P2 with control were performed and the p values are reported.

Supplemental Table 6

	Control	+TTX (1 μM)	+TTX (1 μM) and TTA-P2 (100 nM)
Initial peak voltage change with +50 pA step	6.47 ± 1.17 mV (n=4)	5.40 ± 1.00 mV (n=4, p=0.04)	5.59 ± 1.09 mV (n=4, p=0.04)
Sag amplitude with +50 pA step	1.75 ± 0.37 mV (n=4)	1.69 ± 0.44 mV (n=4, p=0.84)	1.58 ± 0.39 mV (n=4, p=0.24)
R_N with +50 pA step	94.30 ± 24.07 MΩ (n=4)	84.55 ± 19.57 MΩ (n=4, p=0.16)	82.55 ± 23.18 MΩ (n=4, p=0.14)

Supp Table 6 (Related to Fig 6): Effects of inhibiting the Na^+ current with tetrodotoxin in the absence and presence of Ca_V3 currents had little effect on peak voltage, sag and input resistance with small subthreshold depolarizing steps. The effects of 1 μM TTX and when co-applied with TTA-P2 (100 nM) on initial peak voltage changes, sag amplitudes and input resistance when measured using a +50 pA depolarizing step. The measurements were all done at a fixed potential of -70 mV to allow comparison between the different conditions. Paired t-tests comparing the values in the presence of TTX or co-application of TTX and TTA-P2 with control were performed and the p values are reported.

Supplementary Table 7

	50 pA	100 pA	150 pA	200 pA	250 pA	300 pA
Wt ventral vs Cav3.2$^{-/-}$ ventral, RMP, Fig 1c	p=0.9483	p=0.5597	p=0.0092	p<0.0001	p<0.0001	p=0.0078
Wt ventral vs wt dorsal, RMP, Fig 1c	p=0.9422	p=0.5120	p=0.0005	p<0.0001	p<0.0001	p<0.0001
Wt ventral vs Cav3.2$^{-/-}$ dorsal, RMP, Fig 1c	p=0.9477	p=0.5487	p=0.0012	p<0.0001	p<0.0001	p<0.0001
Wt ventral vs Cav3.2$^{-/-}$ ventral, -70 mV, Fig 1d	p>0.9999	p=0.9842	p=0.0315	p<0.0001	p<0.0001	p=0.0003
Wt ventral vs wt dorsal, -70 mV, Fig 1d	p>0.9999	p=0.9825	p=0.0026	p<0.0001	p<0.0001	p<0.0001
Wt ventral vs Cav3.2$^{-/-}$ dorsal, -70 mV, Fig 1d	p>0.9999	p=0.9842	p=0.0125	p<0.0001	p<0.0001	p<0.0001
Cav3.2$^{-/-}$ ventral vs wt dorsal, -70 mV, Fig 1d	p>0.9999	p>0.9999	p=0.5636	p=0.1543	p=0.0416	p=0.0070
Cav3.2$^{-/-}$ ventral vs Cav3.2$^{-/-}$ dorsal, -70 mV, Fig 1d	p>0.9999	p>0.9999	p=0.7490	p=0.2250	p=0.0162	p=0.0021
Wt ventral con vs TTA-P2 (100 nM), Fig 1a	p>0.9999	p>0.9999	p=0.0611	p=0.0273	p=0.0214	p=0.0415

Wt ventral con vs $NiCl_2$ (50 μM), Supp Fig 3	p>0.9999	p>0.9999	p=0.1323	p=0.0314	p=0.0124	p=0.0230
Wt ventral con vs Ril (10 μM), Fig 6a			p=0.0918	p=0.0209	p=0.0095	p=0.0029
$Ca_V3.2^{-/-}$ ventral con vs Ril (10 μM), Fig 6b			p=0.2031	p=0.2376	p=0.2752	p=0.2754
Wt dorsal con vs Ril (10 μM), Fig 6c				p=0.3632	p=0.3006	p=0.054

Supp Table 7: Significance values related to Figs 1, 6 and Supp Fig 3. The table represents the exact p values that were obtained for the plots for action potentials versus current injection in Figs 1, 6 and Supp Fig 3. A two-way ANOVA uncorrected with Fisher's Least Significance Differences was used to obtain p values for Fig 1c and 1d. Paired t-tests were used to obtain p values for Figs 1a, 6 and Supp Fig 3.
